# Building Strategies into QBF Proofs

**DOI:** 10.1007/s10817-020-09560-1

**Published:** 2020-05-22

**Authors:** Olaf Beyersdorff, Joshua Blinkhorn, Meena Mahajan

**Affiliations:** 1 Institut für Informatik, Friedrich-Schiller-Universität Jena, Jena, Germany; 2 The Institute of Mathematical Sciences, HBNI, Chennai, India

**Keywords:** QBF, DQBF, Resolution, Proof complexity

## Abstract

Strategy extraction is of great importance for quantified Boolean formulas (QBF), both in solving and proof complexity. So far in the QBF literature, strategy extraction has been algorithmically performed *from* proofs. Here we devise the first QBF system where (partial) strategies are built *into* the proof and are piecewise constructed by simple operations along with the derivation. This has several advantages: (1) lines of our calculus have a clear semantic meaning as they are accompanied by semantic objects; (2) partial strategies are represented succinctly (in contrast to some previous approaches); (3) our calculus has strategy extraction by design; and (4) the partial strategies allow new sound inference steps which are disallowed in previous central QBF calculi such as Q-Resolution and long-distance Q-Resolution. The last item (4) allows us to show an exponential separation between our new system and the previously studied reductionless long-distance resolution calculus. Our approach also naturally lifts to dependency QBFs (DQBF), where it yields the first sound and complete CDCL-style calculus for DQBF, thus opening future avenues into CDCL-based DQBF solving.

## Introduction

Proof complexity investigates the resources for proving logical theorems, focussing foremost on the minimal size of proofs needed in a particular calculus. Since its inception the field has enjoyed strong connections to computational complexity (cf. [17, 20]) and to first-order logic [19, 38]).

During the past decade, proof complexity has emerged as a key tool to model and analyse advances in the algorithmic handling of hard problems such as SAT and beyond. While traditionally perceived as a computationally hard problem, SAT solvers have been enormously successful in tackling huge industrial instances [42, 56] and hard combinatorial problems [32]. As each run of a solver on an unsatisfiable formula can be understood as a proof of unsatisfiability, each solver implicitly defines a proof system. This connection turns proof complexity into the main theoretical approach towards understanding the power and limitations of solving, with bounds on proof size directly corresponding to bounds on solver running time [17, 43].

The algorithmic success story of solving has not stopped at SAT, but is currently extending to even more computationally complex problems such as *quantified Boolean formulas* (QBF), which is $\mathsf {PSPACE}$ complete, and *dependency QBFs* (DQBF), which is even $\mathsf {NEXP}$ complete [1]. While quantification does not increase expressivity, (D)QBFs can encode many problems far more succinctly, including application domains such as automated planning [18, 22], verification [6, 41], synthesis [24, 40] and ontologies [37].

The past 15 years have seen *huge advances in QBF solving*. While some of the main innovations in SAT solving, including the development of conflict-driven clause learning (CDCL), revolutionised SAT in the late 1990s [53], this development in QBF is happening *now*. Consequently, QBF proof complexity has received considerable attention in recent years.

Compared with QBF, solving in DQBF [26] is at its very beginnings, both in implementations (2018 was the first year that saw a DQBF track in the QBF competition [48]) as well as in its accompanying theory [52].

*Strategy extraction* is one of the distinctive features of QBF and DQBF, manifest in both solving [5, 49] and proof complexity. For solving it guarantees that together with the true/false answer the solver can produce a model (or countermodel) of the (D)QBF. This is an important step in the solving workflow, since a model (or countermodel) may encode a solution (or a counterexample) to the given problem. For example, a model for a QBF encoding a synthesis problem defines an implementation meeting the desired specification [31]. Determining truth merely implies the existence of such a system.

On the proof complexity side, this implies that proof calculi modelling QBF solving should allow strategy extraction in the sense that from a refutation of a false QBF, a countermodel of the QBF can be efficiently constructed. This feature—without analogue in the propositional domain—enables strong lower-bound techniques in QBF proof complexity [9, 11, 12], exploiting the fact that formulas requiring hard strategies cannot have short proofs in calculi with efficient strategy extraction.

As in SAT versus propositional proof complexity, one of the prime challenges in QBF and DQBF is to create compelling proof-theoretic models that capture central features of (D)QBF solving and at the same time remain amenable to a proof-theoretic analysis. While there exist several orthogonal approaches in QBF solving with quite different associated proof calculi, we will focus here on the paradigm of quantified conflict-driven constraint learning (QCDCL) [59]. An interesting feature of QCDCL is that it combines conflict learning with *solution learning*. Whereas a CDCL SAT solver can terminate upon finding a single solution (i.e. a satisfying assignment), a QCDCL QBF solver will repeatedly learn and manipulate solutions, aiming to determine the truth of the input QBF.^1^ Meanwhile, the solver also employs conflict learning, aiming to determine falsity. Here we focus on the conflict learning side. Proof-theoretically its most basic model is Q-Resolution [35], which as in propositional resolution operates on clauses (of prenex QBFs).

*Q-Resolution* ($\mathsf {Q{\hbox {{-}}}Res}$) uses the resolution rule of propositional resolution and augments this with a universal reduction rule that allows to eliminate universal variables from clauses. Combining these two rules requires some technical care: without any side-conditions the two rules result in an unsound system. Typically this is circumvented by prohibiting the derivation of universal tautologies. It was noted early on that in solving this is needlessly prohibitive [59] and universal tautologies can be permitted under certain side-conditions. Later formalised as the proof system *long-distance Q-Resolution* ($\mathsf {LD{\hbox {{-}}}Q{\hbox {{-}}}Res}$) [3], it was even shown that $\mathsf {LD{\hbox {{-}}}Q{\hbox {{-}}}Res}$ exponentially shortens proofs in comparison to $\mathsf {Q{\hbox {{-}}}Res}$ [23], thus demonstrating the appeal of the approach for solving. In fact, when enabling long-distance steps in QBF solving, universal reduction is not strictly needed and this reductionless approach was adopted in the QBF solver GhostQ [36]. To model this solving paradigm, Bjørner, Janota, and Klieber [15] introduced the calculus of *reductionless* $\mathsf {LD{\hbox {{-}}}Q{\hbox {{-}}}Res}$.

The interplay between long-distance resolution and universal reduction steps becomes even more intriguing in DQBF. In [2] it was shown that lifting $\mathsf {Q{\hbox {{-}}}Res}$ (using the rules of resolution and universal reduction) to DQBF results in an incomplete proof system, whereas lifting $\mathsf {LD{\hbox {{-}}}Q{\hbox {{-}}}Res}$ (using long-distance resolution steps together with universal reduction) becomes unsound [13].

Naturally, the intriguing question of why and how deriving ‘universal tautologies’ in long-distance steps might help solving has attracted attention among theoreticians and practitioners alike. Instead of a universal tautology $u\vee {\bar{u}}$, most formalisations of long-distance resolution actually use the concept of a ‘merged’ literal $u^*$. While it is clear (and implicit in the literature) that merged literals $u^*$ correspond to partial strategies for *u* rather than universal tautologies, a formal semantic account of long-distance steps (and stronger calculi using merging [12]) was only recently given by Suda and Gleiss [54], where partial strategies are constructed for each individual proof inference. However, as already noted in [54], the models considered in [54] fail to have efficient strategy extraction in the sense that the constructed (partial) strategies may need exponential-size representations.

### Our contributions

**A. The new calculus of Merge Resolution.** Starting from the reductionless $\mathsf {LD{\hbox {{-}}}Q{\hbox {{-}}}Res}$ system of [15] and its role of modelling QCDCL solving, we develop a new calculus that we call Merge Resolution ($\mathsf {M{\hbox {{-}}}Res}$). Like reductionless $\mathsf {LD{\hbox {{-}}}Q{\hbox {{-}}}Res}$, the system $\mathsf {M{\hbox {{-}}}Res}$ only uses a resolution rule and does not permit universal reduction steps. Reductionless $\mathsf {LD{\hbox {{-}}}Q{\hbox {{-}}}Res}$ and $\mathsf {M{\hbox {{-}}}Res}$ are therefore both refutational calculi that finish as soon as they derive a purely universal clause.

As the prime novel feature of $\mathsf {M{\hbox {{-}}}Res}$ we build partial strategies *into* proofs. We achieve this by computing explicit representations of strategies in a variant of binary decision diagrams (called *merge maps* here), which are updated and refined at each proof step by simple operations. These merge maps are part of the proof. As a consequence, $\mathsf {M{\hbox {{-}}}Res}$ has efficient strategy extraction by design.

This is in contrast to all previous existing QBF calculi in the literature, where strategies are algorithmically constructed *from* proofs. In particular, this also applies to the approaches taken in [23, 54] for $\mathsf {LD{\hbox {{-}}}Q{\hbox {{-}}}Res}$ and in [15] for reductionless $\mathsf {LD{\hbox {{-}}}Q{\hbox {{-}}}Res}$. But also the choice of our representation as merge maps matters: as [15, 54] both represent (partial) strategies as trees, the constructed strategies may grow exponentially in the proof size, thus losing the property of efficient strategy extraction desired for practice. In contrast, in our model merge maps are always linear in the size of the clause derivations.

**B. Exponential separation of** $\mathsf {M{\hbox {{-}}}Res}$ **from reductionless** $\mathsf {LD{\hbox {{-}}}Q{\hbox {{-}}}Res}$. Including merge maps explicitly into proofs also has another far-reaching advantage: it allows resolution steps not only forbidden in $\mathsf {Q{\hbox {{-}}}Res}$, but even disallowed in $\mathsf {LD{\hbox {{-}}}Q{\hbox {{-}}}Res}$. In a nutshell, $\mathsf {LD{\hbox {{-}}}Q{\hbox {{-}}}Res}$ allows resolution steps only when universal variables quantified left of the pivot have *constant and equal* strategies in both parent clauses. In $\mathsf {M{\hbox {{-}}}Res}$ we have explicit representations of strategies and thus can allow resolution steps as long as the strategies in both parent clauses are *isomorphic* to each other, a property that we can check efficiently for merge maps.

This last mentioned advantage of allowing resolution steps in $\mathsf {M{\hbox {{-}}}Res}$ forbidden in (reductionless) $\mathsf {LD{\hbox {{-}}}Q{\hbox {{-}}}Res}$ manifests in shorter proofs. We show this by explicitly giving an example of a family of QBFs that admit linear-size proofs in $\mathsf {M{\hbox {{-}}}Res}$ (Theorem 29), but require exponential size in reductionless $\mathsf {LD{\hbox {{-}}}Q{\hbox {{-}}}Res}$ (Theorem 28). The separating formulas are a variant of the equality formulas introduced in [9]. While the original formulas from [9] are hard for $\mathsf {Q{\hbox {{-}}}Res}$, but easy in $\mathsf {LD{\hbox {{-}}}Q{\hbox {{-}}}Res}$, we here consider a ‘squared’ version, for which we naturally use resolution steps for clauses with associated non-constant winning strategies, allowed in $\mathsf {M{\hbox {{-}}}Res}$, but forbidden in $\mathsf {LD{\hbox {{-}}}Q{\hbox {{-}}}Res}$.

This demonstrates that $\mathsf {M{\hbox {{-}}}Res}$ is exponentially stronger than reductionless $\mathsf {LD{\hbox {{-}}}Q{\hbox {{-}}}Res}$, thus also pointing towards potential improvements in QCDCL solving. While the simulation of reductionless $\mathsf {LD{\hbox {{-}}}Q{\hbox {{-}}}Res}$ by $\mathsf {M{\hbox {{-}}}Res}$ is almost immediate and also the upper bound in $\mathsf {M{\hbox {{-}}}Res}$ is comparatively straightforward, the lower bound is a technically involved argument specifically tailored towards the squared equality formulas.

**C. A sound and complete CDCL-style calculus for DQBF.** As our final contribution we show that the new QBF system of $\mathsf {M{\hbox {{-}}}Res}$ naturally lifts to a sound and complete calculus for DQBF. As shown in [2], the lifting of $\mathsf {Q{\hbox {{-}}}Res}$ to DQBF is incomplete, whereas the combination of universal reduction and long-distance steps presents soundness issues, both in DQBF [13] as well as in the related framework of dependency schemes [7, 8].

Here we show that our framework of $\mathsf {M{\hbox {{-}}}Res}$ overcomes both these soundness and completeness issues and therefore has exactly the right strength for a natural DQBF resolution calculus. In fact, it is the first DQBF CDCL-style system in the literature^2^ and as such paves the way towards CDCL-style solving in DQBF. Again, by design our DQBF system has efficient strategy extraction.

## Preliminaries

*Propositional logic* Let ${\mathcal {Z}}$ be a countable set of Boolean variables. A *literal* is a Boolean variable $z \in {\mathcal {Z}}$ or its negation ${\bar{z}}$, a *clause* is a set of literals, and a *CNF* is a set of clauses. For a literal *l*, we define $\hbox { var}(l) := z$ if $l = z$ or $l = {\bar{z}}$; for a clause *C*, we define $\hbox { vars}(C) := \{\hbox { var}(l) : l \in C\}$; for a CNF $\phi$ we define $\hbox { vars}(\phi ) := \cup _{C \in \phi } \hbox { vars}(C)$.

An assignment to a set $Z \subseteq {\mathcal {Z}}$ of Boolean variables is a function $\rho : Z \rightarrow \{0,1\}$, conventionally represented as a set of literals in which *z* (resp. ${\bar{z}}$) represents the assignment $z \mapsto 1$ (resp. $z \mapsto 0$). The set of all assignments to *Z* is denoted $\langle Z \rangle$. Given a subset $Z^\prime \subseteq Z$, $\rho {\upharpoonright _{Z^\prime }}$ is the restriction of $\rho$ to $Z^\prime$. The CNF $\phi [\rho ]$ is obtained from $\phi$ by removing any clause containing a literal in $\rho$, and removing the negated literals $\{{\bar{l}} : l \in \rho \}$ from the remaining clauses. We say that $\rho$ *falsifies* $\phi$ if $\phi [\rho ]$ contains the empty clause, and that $\phi$ is *unsatisfiable* if it is falsified by each $\rho \in \langle Z \rangle$.

Given two clauses $R_1$ and $R_2$ and a literal *l* such that $l \in R_1$ and ${\bar{l}} \in R_2$, we define the resolvent $\hbox { res}(R_1,R_2,l) := (R_1 {\setminus } \{l\}) \cup (R_2 {\setminus } \{{\bar{l}}\})$. (Note that $\hbox { res}(R_1,R_2,l) = \hbox { res}(R_2,R_1,{\bar{l}})$.) A *resolution refutation* of a CNF $\phi$ is a sequence $C_1, \dots ,C_k$ of clauses in which $C_k$ is the empty clause and, for each $i \in [k]$, either (a) $C_i \in \phi$ or (b) $C_i = \hbox { res}(C_a,C_b,z)$ for some $a,b < i$ and $z \in \hbox { vars}(\phi )$.

*Quantified Boolean formulas* A *quantified Boolean formula* (QBF) in *prenex conjunctive normal form* (PCNF) is denoted $\varPhi :={\mathcal {Q}}\cdot {\phi }$, where (a) $\mathcal {Q}:= \mathcal {Q}_1 Z_1 \cdots \mathcal {Q}_n Z_n$ is the *quantifier prefix*, in which the $Z_i \subset {\mathcal {Z}}$ are pairwise disjoint finite sets of Boolean variables, $\mathcal {Q}_i \in \{\exists , \forall \}$ for each $i \in [n]$, and $\mathcal {Q}_i \ne \mathcal {Q}_{i+1}$ for each $i \in [n-1]$, and (b) the *matrix* $\phi$ is a CNF over $\hbox { vars}(\varPhi ) := \bigcup ^n_{i=1} Z_i$.

The existential (resp. universal) variables of $\varPhi$, typically denoted *X* (resp. *U*), is the set obtained as the union of the $Z_i$ for which $\mathcal {Q}_i = \exists$ (resp. $\mathcal {Q}_i = \forall$). The prefix $\mathcal {Q}$ defines a binary relation $<_\mathcal {Q}$ on $\hbox { vars}(\varPhi )$, such that $z <_\mathcal {Q}z^\prime$ holds iff $z \in Z_i$, $z^\prime \in Z_j$, and $i<j$, in which case we say that $z^\prime$ *is right of* *z* and *z* *is left of* $z^\prime$. For each $u \in U$, we define $L_\mathcal {Q}(u) := \{x \in X : x <_\mathcal {Q}u\}$, i.e. the existential variables left of *u*.

*QBF semantics* Semantics for QBFs is neatly described by the *two-player evaluation game*. Over the course of a game, the variables of a QBF ${\mathcal {Q}}\cdot {\phi }$ are assigned 0/1 values in the order of the prefix, with the $\exists$-player ($\forall$-player) choosing the values for the existential (universal) variables. When the game concludes, the players have constructed a total assignment $\rho$ to the variables. The $\forall$-player wins iff $\rho$ falsifies $\phi$.

A *strategy* dictates how the $\forall$-player should respond to every possible move of the $\exists$-player. A strategy *h* for a QBF $\varPhi$ is a set $\{h_u : u \in U\}$ of functions $h_u : \langle L_\mathcal {Q}(u) \rangle \rightarrow \{u, {\bar{u}}\}$. Additionally *h* is *winning* if, for each $\alpha \in \langle X \rangle$, the restriction of $\phi$ by $\alpha \cup \{h_u(\alpha {\upharpoonright _{L_\mathcal {Q}(u)})} : u \in U\}$ contains the empty clause. We use the terms ‘winning strategy’ and ‘countermodel’ interchangeably. A QBF is called *false* if it has a countermodel, and *true* if it does not.

A *partial strategy* for a universal variable *u* is a function from some subset of $\langle L_\mathcal {Q}(u) \rangle$ into $\{u, {\bar{u}}\}$.

*QBF proof systems* We deal with line-based refutational QBF systems that typically employ axioms and inference rules to prove the falsity of QBFs. We say that $\mathsf {P}$ is *complete* if there exists a $\mathsf {P}$ refutation of every false QBF, *sound* if there exists no $\mathsf {P}$ refutation of any true QBF. We call $\mathsf {P}$ a *proof system* if it is sound, complete, and polynomial-time checkable. Given two QBF proof systems $\mathsf {P}_1$ and $\mathsf {P}_2$, $\mathsf {P}_1$ *p-simulates* $\mathsf {P}_2$ if there exists a polynomial-time procedure that takes a $\mathsf {P}_{2}$-refutation and outputs a $\mathsf {P}_{1}$-refutation of the same QBF [20].

## Reductionless long-distance Q-Resolution

**Fig. 1:**
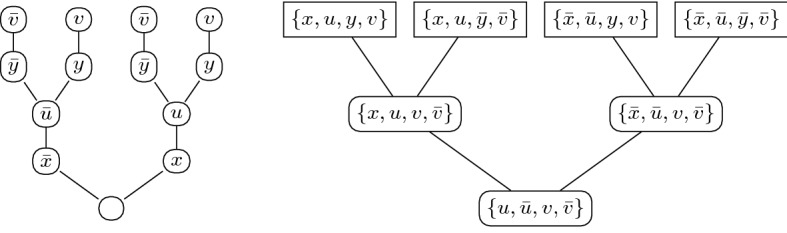
The full binary tree depiction of a countermodel and its associated reductionless $\mathsf {LD{\hbox {{-}}}Q{\hbox {{-}}}Res}$ refutation

In this section we recall the definition of reductionless $\mathsf {LD{\hbox {{-}}}Q{\hbox {{-}}}Res}$, prove that it is refutationally complete, and demonstrate that it does not have polynomial-time strategy extraction in either of the computational models of [15, 54]. The system appeared first in [15, Fig. 1], where it was referred to as $Q^w$-resolution.

### Definition 1

(*reductionless* $\mathsf {LD{\hbox {{-}}}Q{\hbox {{-}}}Res}$ [15]) In reductionless $\mathsf {LD{\hbox {{-}}}Q{\hbox {{-}}}Res}$, a *derivation* from a QBF $\varPhi := {\mathcal {Q}}\cdot {\phi }$ is a sequence $\pi := C_1, \dots ,C_k$ of clauses in which at least one of (a) or (b) holds for each $i \in [k]$:

- **Axiom.** $C_i$ is a clause from the matrix $\phi$;
- **Long-distance resolution.** There exist integers $a,b < i$ and an existential pivot $x \in X$ such that $C_i = \hbox { res}(C_a,C_b,x)$ and, for each $u \in \hbox { vars}_\forall (C_a) \cap \hbox { vars}_\forall (C_b)$, if $u <_\mathcal {Q}x$, then $\{u,{\bar{u}}\} \nsubseteq C_i$.

The final clause $C_k$ is the *conclusion* of $\pi$, and $\pi$ is a *refutation* of $\varPhi$ iff $C_k$ contains no existential variables.

A pair of complementary universal literals $\{u,{\bar{u}}\}$ appearing in a clause is referred to singly as a *merged literal*. It is clear from a wealth of literature^3^ that merged literals are ‘placeholders’ for partial strategies, the exact representation left implicit in the structure of the derivation.

We illustrate the rules of the calculus by showing that the equality formulas [9] have linear-size refutations.

### Definition 2

(*equality formulas* [9]) The *equality family* is the QBF family whose $n^{\hbox {{th}}}$ instance has the prefix $\exists \{x_1, \dots , x_n\} \forall \{u_1, \dots , u_n\} \exists \{t_1, \dots ,t_n \}$ and the matrix consisting of the clauses $\{x_i,u_i,t_i\},\{{\bar{x}}_i,{\bar{u}}_i,t_i\}$ for $i \in [n]$, and $\{{\bar{t}}_1, \dots ,{\bar{t}}_n\}$.

### Example 3

We construct linear-size reductionless $\mathsf {LD{\hbox {{-}}}Q{\hbox {{-}}}Res}$ refutations in two stages. First, resolve each pair $\{x_i,u_i,t_i\}$, $\{{\bar{x}}_i, {\bar{u}}_i, t_i\}$ of clauses over pivot $x_i$ to obtain $C_i := \{u_i,{\bar{u}}_i,t_i\}$. Note that it is allowed to introduce the merged literal $\{u_i, {\bar{u}}_i\}$ since variable $u_i$ is right of the pivot $x_i$. Second, resolve the $C_i$ successively against the long clause $\{{\bar{t}}_1, \dots , {\bar{t}}_n\}$ over pivot $t_i$, to obtain a full set of merged literals $C := \{u_i, {\bar{u}}_i : i \in [n]\}$. Here, even though $u_i$ is left of the pivot $t_i$, the appearance of the merged literal $\{u_i,\bar{u_i}\}$ in the resolvent is allowed, since variable $u_i$ is absent from one of the antecedents. The derivation is a refutation since the conclusion *C* contains no existential literals.

We now show that this calculus is indeed complete. Given a false QBF $\varPhi$ with a countermodel *h*, we construct a canonical reductionless $\mathsf {LD{\hbox {{-}}}Q{\hbox {{-}}}Res}$ refutation based on the ‘full binary tree’ representation of a countermodel [51]. For each $\alpha \in \langle X \rangle$, there exists some $C_\alpha$ in the matrix falsified by $\alpha \cup h(\alpha )$. The set of all such $C_\alpha$ may be successively resolved over existential pivots in reverse prefix order, finally producing a clause containing no existentials. Merged literals never block resolution steps in this construction, as they only ever appear to the right of the pivot variable.

### Example 4

Consider the QBF with the prefix $\exists \{x\} \forall \{u\} \exists \{y\} \forall \{v\}$ and the matrix consisting of the clauses

$$\begin{aligned} \{x, u, y, v\}, \{x, u, {\bar{y}}, {\bar{v}}\}, \{{\bar{x}}, {\bar{u}}, y, v\}, \{{\bar{x}}, {\bar{u}}, {\bar{y}}, {\bar{v}}\}\,. \end{aligned}$$

It is easy to see that the unique countermodel for this QBF essentially sets *u* and *v* equal to *x* and *y*, respectively. Formally, the countermodel consists of the functions $h_u$ and $h_v$, where $h_u(\alpha )(u) = \alpha (x)$ and $h_v(\beta )(v) = \beta (y)$, for each $\alpha \in \langle \{x\} \rangle$ and $\beta \in \langle \{x,y\} \rangle$.

Figure 1 shows the full binary tree depiction of this countermodel and its associated reductionless $\mathsf {LD{\hbox {{-}}}Q{\hbox {{-}}}Res}$ refutation. Notice that each path from root to leaf in the countermodel tree specifies a total assignment that falsifies the corresponding axiom clause. Notice also that the existential resolution pivots on each path from an axiom to the conclusion occur in reverse prefix order, matching the pattern of the full binary tree countermodel. The prefix order inherent to the countermodel tree also ensures that each long-distance resolution step is valid.

### Lemma 5

Every false QBF has a reductionless $\mathsf {LD{\hbox {{-}}}Q{\hbox {{-}}}Res}$ refutation.

### Proof

Let $\varPhi := {\mathcal {Q}}\cdot {\phi }$ be a false QBF with countermodel *h*. Let $\{x_1, \dots , x_n\}$ denote the existential variables of $\varPhi$ in prefix order; that is, for each $i,j \in [n]$ with $i < j$, $x_i$ is not right of $x_j$. Let $\alpha _1, \dots , \alpha _{2^n}$ define the natural lexicographic ordering of the total assignments to *X*, as inWe define a sequence $\pi := \pi _n \circ \dots \circ \pi _0$ in which each $\pi _i := C^i_1, \dots ,C^i_{2^i}$, and the clauses $C^i_j$ are defined recursively as follows: For $j \in [2^n]$, $C^n_j$ is any clause in $\phi$ falsified by $\alpha _j \cup h(\alpha _j)$ (at least one such clause exists by definition of countermodel); for $i \in [n]$ and $j \in [2^{i-1}]$, $C^{i-1}_j := \hbox { res}(C^i_{2j-1},C^i_{2j},x_i)$ if this resolvent exists, otherwise

$$\begin{aligned} C^{i-1}_j := {\left\{ \begin{array}{ll} C^i_{2j-1}\,,&{}\text{ if } x_i \notin C^i_{2j-1}\,,\\ C^i_{2j}\,,&{}\text{ if } {\bar{x}}_i \notin C^i_{2j}\,. \end{array}\right. } \end{aligned}$$

It is readily verified by downwards induction on $i \in [n]$ that each $C^i_j$ contains no complementary universal literals in variables left of $x_i$. Moreover, it is easy to see that the conclusion $C^0_1$ contains no existential literals. Removing duplicate clauses from $\pi$ produces a reductionless $\mathsf {LD{\hbox {{-}}}Q{\hbox {{-}}}Res}$ refutation of $\varPhi$. $\square$

Soundness and polynomial-time checkability of reductionless $\mathsf {LD{\hbox {{-}}}Q{\hbox {{-}}}Res}$ are immediate, as the system uses a subset of the rules of the classical long-distance Q-resolution proof system [3].

*The computational model of Bjørner et al.* [15]. In tandem with reductionless $\mathsf {LD{\hbox {{-}}}Q{\hbox {{-}}}Res}$, the authors of [15] introduced a computational model based on tree-like branching programs. The model is used to explicitly construct the partial strategies represented implicitly by merged literals.

We demonstrate that tree-like branching programs constructed in this way cannot represent strategies efficiently; that is, the system does not have polynomial-time strategy extraction in the associated model (even for partial strategies). The following example shows a linear-size derivation whose explicit strategy grows exponentially large.

### Example 6

Consider the following proof fragment, in reductionless $\mathsf {LD{\hbox {{-}}}Q{\hbox {{-}}}Res}$, with a prefix $\exists v \exists x \exists w \forall u \exists y \exists z$. Alongside each proof line is the strategy for the universal variable *u*, as built by the Build function in [15]. In a nutshell, Build traverses the subderivation of the current step, and represents the pattern of merges on *u* as a tree-like branching program that queries the (existential) resolution pivots.

**Table:** 

Line	Obtained as	Clause	Strategy as built in [15]
$C_1$	axiom	$\{w, x, u\}$	0
$C_2$	axiom	$\{{\bar{w}} , x , {\bar{u}}\}$	1
$C_3$	$\hbox { res}(C_1,C_2,w)$	$\{x , u , {\bar{u}}\}$	$w \mathbin {?}1:0$
$C_4$	axiom	$\{{\bar{x}} , u , y\}$	0
$C_5$	$\hbox { res}(C_3,C_4,x)$	$\{u , {\bar{u}} , y\}$	$x \mathbin {?}0:[w \mathbin {?}1:0]$
$C_6$	axiom	$\{v , {\bar{y}}\}$	$*$
$C_7$	$\hbox { res}(C_5,C_6,y)$	$\{v , u , {\bar{u}}\}$	$x \mathbin {?}0:[w \mathbin {?}1:0]$
$C_8$	axiom	$\{{\bar{x}} , z\}$	$*$
$C_9$	$\hbox { res}(C_3,C_8,x)$	$\{u , {\bar{u}} , z\}$	$w \mathbin {?}1:0$
$C_{10}$	axiom	$\{{\bar{v}} , {\bar{z}}\}$	$*$
$C_{11}$	$\hbox { res}(C_9,C_{10},z)$	$\{{\bar{v}} , u , {\bar{u}}\}$	$w \mathbin {?}1:0$
$C_{12}$	$\hbox { res}(C_{7},C_{11},v)$	$\{u , {\bar{u}}\}$	$v \mathbin {?}(w \mathbin {?}1:0):(x \mathbin {?}0:[w \mathbin {?}1:0])$

Observe that the final strategy at line 12 represents the strategy corresponding to line 3 twice. By nesting such a proof fragment from lines $C_3$ to $C_{12}$ with fresh copies of the existential variables (*v*, *x*, *y*, *z*) *k* times, we can construct a reductionless $\mathsf {LD{\hbox {{-}}}Q{\hbox {{-}}}Res}$ proof fragment with *O*(*k*) lines, where the strategy built by the Build function from [15] has size exponential in *k*.

*The computational model of Suda and Gleiss* [54].  The authors of [54] proposed a model of partial strategies based on so-called *policies* (a policy is a set of assignments specifying an ordered decision tree.) They noted that the equality formulas have linear-size refutations in the strong QBF system $\mathsf {IRM{\hbox {{-}}}calc}$ [12], whereas policies witnessing their falsity must be exponentially large, therefore $\mathsf {IRM{\hbox {{-}}}calc}$ does not admit polynomial-time strategy in policies. The same is true for reductionless $\mathsf {LD{\hbox {{-}}}Q{\hbox {{-}}}Res}$, since Example 3 shows that the equality formulas also have linear-size refutations there.

The computational model of policies is not even suitable for strategy extraction in the weak system level-ordered $\mathsf {Q{\hbox {{-}}}Res}$ [34].^4^ Versions of the equality formulas in which the prefix is rearranged ($\exists x_1 \forall u_1 \exists t_1 \cdots \exists x_n \forall u_n \exists t_n$) have linear-size level-ordered $\mathsf {Q{\hbox {{-}}}Res}$ refutations, whereas winning strategies represented as policies must be large. The argument is the same as for the equality formulas [54], and derives from the implicit use of tree-like structures.

That neither model is suitable for efficient strategy extraction shows that using either *inside* the derivation would result in an artificial, exponential size blow-up. The root of the issue is tree-like models versus DAG-like proofs. The DAG-like computational model that we introduce in the following section is tightly knitted to the refutation, yielding linear-time strategy extraction for free.

## Merge resolution

In this section we introduce Merge Resolution ($\mathsf {M{\hbox {{-}}}Res}$, Sect. 4.2), and prove that it is sound and complete for QBF (Sect. 4.3). The salient feature of $\mathsf {M{\hbox {{-}}}Res}$ is the built-in partial strategies, represented as *merge maps*. Given the problems with the computational models of [15, 54], the principal technical challenge is to find a suitable way to define and combine partial strategies devoid of an artificial proof-size inflation.

### Merge maps

*Our computational model* A merge map is a branching program that queries a set of existential variables and outputs an assignment to some universal variable, i.e. a literal in $\{u, {\bar{u}}, *\}$, where $*$ stands for ‘no assignment’. As we intend to tie the DAG structure of the merge maps to the DAG structure of the proof, we will label query nodes with natural numbers based on the proof line indexing (we elaborate on this later). Hence, from a technical standpoint it makes sense to define a merge map as a function from the index set of its nodes.

#### Definition 7

(*merge map*) A *merge map* *M* for a Boolean variable *u* over a finite set *X* of Boolean variables is a function from a finite set *N* of natural numbers satisfying, for each $i \in N$, either $M(i) \in \{u,{\bar{u}},*\}$ or $M(i) \in X \times N_{<i} \times N_{<i}$, where $N_{<i} := \{i^\prime \in N : i^\prime < i\}$.

A triple of the form $(x,a,b) \in X \times N_{<i} \times N_{<i}$ represents the instruction ‘if $x = 0$ then goto *a* else goto *b*’, whereas the literals $\{u,{\bar{u}},*\}$ represent output values. The exact computation is formalised below.

#### Definition 8

(*computed function*) Let *M* be a merge map for *u* over *X* with domain *N*. The *function computed by M* is the function

$$\begin{aligned} h: \langle X \rangle \rightarrow \{u,{\bar{u}},*\} \end{aligned}$$

mapping $\alpha \in \langle X \rangle$ to the output of the following algorithm:

1. $$i := \max (N)$$
2. $$\mathbf{while } M(i) \notin \{u,{\bar{u}},*\}$$
3. $$(x,a,b) := M(i)$$
4. $$\mathbf{if } {\bar{x}} \in \alpha \mathbf{ then } i := a \mathbf{ else } i:=b$$
5. $$\mathbf{return } M(i)$$

We depict merge maps pictorially as DAGs. The nodes are the domain elements, and the leaf nodes as well as the directed edges are labelled by literals. In a merge map *M*, if *M*(*i*) is a literal *l*, then node *i* is labeled *l*. If $M(i) = (x,a,b)$, then the DAG has the edge $i \rightarrow a$ labeled ${\bar{x}}$ and the edge $i\rightarrow b$ labeled *x*. The DAG naturally describes a deterministic branching program computing a Boolean function.

**Fig. 2:**
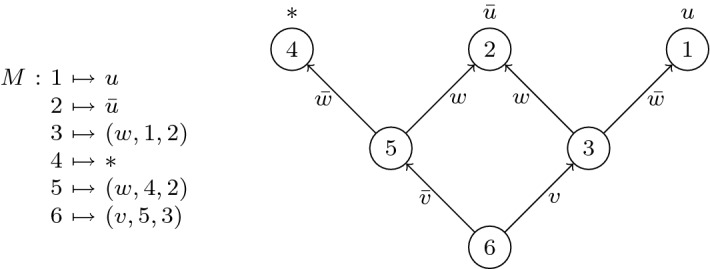
Function and branching program representations of a merge map *M*

Figure 2 shows a merge map represented as a function, and its corresponding depiction as a branching program.

*Relations* Merge Resolution uses two relations to determine preconditions for the binary operations. Firstly, we give $\mathsf {M{\hbox {{-}}}Res}$ the power to identify merge maps with equivalent representations, up to indexing. We term equivalent representations ‘isomorphic’.

#### Definition 9

(*isomorphism*) Two merge maps $M_1$ and $M_2$ for *u* over *X* with domains $N_1$ and $N_2$ are *isomorphic* (written $M_1 \simeq M_2$) iff there exists a bijection $f:N_1 \rightarrow N_2$ such that the following hold for each $i \in N_1$:

- if $M_1(i)$ is a literal in $\{u,{\bar{u}},*\}$ then $M_2(f(i)) = M_1(i)$;
- if $M_1(i)$ is the triple (*x*, *a*, *b*) then $M_2(f(i)) = (x,f(a),f(b))$.

#### Proposition 10

Any two isomorphic merge maps compute the same function.

#### Proof

Let $M_1$ and $M_2$ be merge maps, let *f* be a bijection satisfying the properties of Definition 9, and let $i \in \hbox { dom}(M_1)$. The computation of $M_2(i)$ as in Definition 8 is identical to that of $M_1$, except that each natural number $a \in \hbox { dom}(M_1)$ is replaced with *f*(*a*). The proposition follows. $\square$

Our second relation, consistency, simply identifies whether or not two merge maps agree on the intersection of their domains.

#### Definition 11

(*consistency*) Two merge maps $M_1$ and $M_2$ for *u* over *X* with domains $N_1$ and $N_2$ are *consistent* (written $M_1 \bowtie M_2$) iff $M_1(i) = M_2(i)$ for each $i \in N_1 \cap N_2$.

#### Example 12

**Fig. 3:**
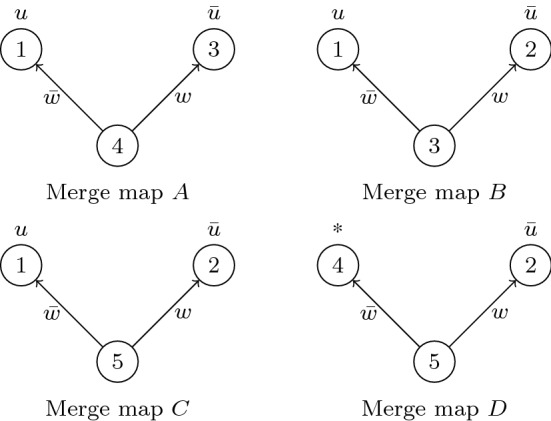
Relations on merge maps

For the merge maps depicted in Fig. 3, isomorphism and consistency (or lack thereof) are as given in the table below.

**Table:** 

Relation	Isomorphic	Not isomorphic
Consistent	$A\bowtie C$; $A \simeq C$	$B\bowtie D$; $B\not \simeq D$
Not consistent	$A\not \bowtie B$; $A\simeq B$	$C\not \bowtie D$; $C\not \simeq D$

It is easy to see that both relations can be computed in time polynomial in $\max (N_1 \cup N_2)$. (To check isomorphism, step through the two merge maps starting from their maximal domain elements $N_1, N_2$. Using memoization, iteratively build the bijection-witnessing isomorphism. Any suitable data structure that allows efficient insertion and search can be used for this. To check consistency, construct the two domains—again, using an appropriate data structure, and check that the instructions at common line numbers match.)

*Operations* $\mathsf {M{\hbox {{-}}}Res}$ uses two binary operations to build merge maps for the resolvent based on those of the antecedents. We define the operations and give some intuition on their role in $\mathsf {M{\hbox {{-}}}Res}$. Concrete examples follow the definition of the system in the next subsection.

The *select* operation identifies equivalent merge maps by means of the isomorphism relation. It also allows a *trivial* merge map to be discarded; we call a merge map trivial iff it is isomorphic to $1 \mapsto *$. (The operation is undefined if the merge maps are neither isomorphic nor do they contain a trivial map.)

#### Definition 13

(*select*) Let $M_1$ and $M_2$ be merge maps for which $M_1 \simeq M_2$ or one of $M_1, M_2$ is trivial. Then $\hbox { select}(M_1,M_2) := M_2$ if $M_1$ is trivial, and $\hbox { select}(M_1,M_2) := M_1$ otherwise.

The *merge* operation allows two consistent merge maps to be combined as the children of a fresh query node. Antecedent maps are only ever merged for universal variables right of the pivot *x*. The inclusion of a natural number *n* allows the new query node to be identified with the resolvent, via its index in the proof sequence. In this way, query nodes are shared between later merge maps, rather than being duplicated; the result is a DAG-like structure which faithfully follows that of the derivation.

#### Definition 14

(*merge*) Let $M_1$ and $M_2$ be consistent merge maps for *u* over *X* with domains $N_1$ and $N_2$, let $n > \max (N_1 \cup N_2)$ be a natural number, and let $x \in X$. Then $\hbox { merge}(M_1,M_2,n,x)$ is the function from $N_1 \cup N_2 \cup \{n\}$ defined by

$$\begin{aligned} \hbox { merge}(M_1,M_2,n,x)(i) := {\left\{ \begin{array}{ll} (x,\max (N_1),\max (N_2))&{}\text{ if } i = n,\\ M_1(i)&{}\text{ if } i \in N_1,\\ M_2(i)&{}\text{ if } i \in N_2 {\setminus } N_1. \end{array}\right. } \end{aligned}$$

#### Example 15

In Fig. 3, we have $\hbox { select}(A,B)=\hbox { select}(A,C)=A$. Also, $\hbox { merge}(D,B,6,v)$ gives the merge map from Fig. 2.

### Definition of $\mathsf {M{\hbox {{-}}}Res}$

We are now ready to put down the rules of Merge Resolution. Given a non-tautological clause *C* and a Boolean variable *u*, the *falsifying u-literal for C* is ${\bar{l}}$ if there is a literal $l \in C$ with $\hbox { var}(l) = u$, and $*$ otherwise.

#### Definition 16

(*merge resolution*) Let $\varPhi := {\mathcal {Q}}\cdot {\phi }$ be a QBF with existential variables *X* and universal variables *U*. A *merge resolution* ($\mathsf {M{\hbox {{-}}}Res}$) *derivation* of $L_k$ from $\varPhi$ is a sequence $\pi := L_1, \dots , L_k$ of lines $L_i := (C_i,\{M^u_i: u \in U\})$ in which at least one of the following holds for each $i \in [k]$:

- **Axiom.** There exists a clause in $C \in \phi$ such that $C_i$ is the existential subclause of *C*, and, for each $u \in U$, $M^u_i$ is the merge map for *u* over $L_\mathcal {Q}(u)$ with domain $\{i\}$ mapping *i* to the falsifying *u*-literal for *C*;
- **Resolution.** There exist integers $a,b < i$ and an existential pivot $x \in X$ such that $C_i = \hbox { res}(C_a,C_b,x)$ and, for each $u \in U$, either
  (i) $$M^u_i = \hbox { select}(M^u_a,M^u_b)$$, or
  (ii) $$x <_\mathcal {Q}u$$ and $$M^u_i = \hbox { merge}(M^u_a, M^u_b ,i, x)$$.

The final line $L_k$ is the *conclusion* of $\pi$, and $\pi$ is a *refutation* of $\varPhi$ iff $C_k = \emptyset$. The *size* of $\pi$ is $|\pi | = k$.

Note that the order of the indexes *a* and *b* in $\hbox { merge}(M_a,M_b,i,x)$ matches that of $\hbox { res}(C_a,C_b,x)$. This is why we interpret the triple (*x*, *a*, *b*) as ‘if $x=0$ then goto *a* else goto *b*’. Using the conventional ‘if $x=1$’ entails swapping the order of the arguments $M_a$ and $M_b$.

We illustrate the rules of $\mathsf {M{\hbox {{-}}}Res}$ with two examples. The first demonstrates that labelling branching nodes with proof-line indexes sidesteps the exponential blow-up in the computational model of [15].

**Fig. 4:**
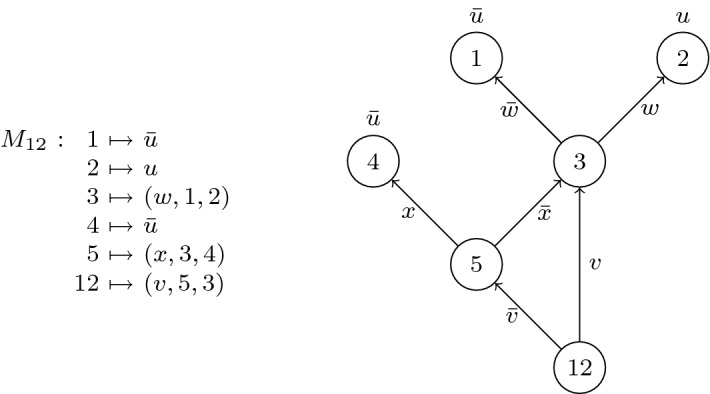
Function and branching program representations of $M_{12}$ from Example 17

#### Example 17

The reductionless $\mathsf {LD{\hbox {{-}}}Q{\hbox {{-}}}Res}$ proof fragment in Example 6 can be viewed as a proof in $\mathsf {M{\hbox {{-}}}Res}$ if we attach appropriate merge maps at each line.

**Table:** 

Line	Rule	$C_i$	$M_i$	Query
$L_1$	axiom	$\{w, x \}$	$1\mapsto {\bar{u}}$	
$L_2$	axiom	$\{{\bar{w}}, x \}$	$2\mapsto u$	
$L_3$	$\hbox { res}(L_1,L_2,w)$	$\{x\}$	$\hbox { merge}(M_1,M_2,3,w)$	$3 \mapsto (w,1,2)$
$L_4$	axiom	$\{{\bar{x}}, y\}$	$4 \mapsto {\bar{u}}$	
$L_5$	$\hbox { res}(L_3,L_4,x)$	$\{ y \}$	$\hbox { merge}(M_3,M_4,5,x)$	$5 \mapsto (x,3,4)$
$L_6$	axiom	$\{v,{\bar{y}}\}$	$6 \mapsto *$	
$L_7$	$\hbox { res}(L_5,L_6,y)$	$\{v\}$	$\hbox { select}(M_5,M_6) = M_5$	
$L_8$	axiom	$\{{\bar{x}}, z\}$	$8 \mapsto *$	
$L_9$	$\hbox { res}(L_3,L_8,x)$	$\{z\}$	$\hbox { select}(M_3,M_8) = M_3$	
$L_{10}$	axiom	$\{{\bar{v}}, {\bar{z}}\}$	$10 \mapsto *$	
$L_{11}$	$\hbox { res}(L_9,L_{10},z)$	$\{{\bar{v}}\}$	$\hbox { select}(M_9,M_{10}) =$	
			$$\hbox { select}(M_3,M_{10}) = M_3$$	
$L_{12}$	$\hbox { res}(L_7,L_{11},v)$	$\{\}$	$\hbox { merge}(M_{7},M_{11},12,v)$	$12 \mapsto (v,5,3)$
			$$= \hbox { merge}(M_5,M_3,12,v)$$	

In lines $L_7$, $L_9$ and $L_{11}$, the use of $\hbox { select}$ is allowed, since in each case one of the antecedent merge maps is trivial (i.e. isomorphic to $1 \mapsto *$). Notice that at line $L_7$, we could also have chosen $M_7$ to be $\hbox { merge}(M_5,M_6,7,y)$; this would result in a larger merge map.

Now, consider the final merge map $M_{12}$. The corresponding branching program has isolated nodes numbered 6, 8, and 10; these can be removed, giving the pruned merge map shown in Fig. 4. Notice how the size blow-up from Example 6 is avoided here; since $M_3$ and $M_5$ are consistent, node 12 simply points to both of them, and the shared part (that is, the branching program $M_3$ containing nodes 1, 2, and 3) is represented just once.

Our second example illustrates how the explicit representation of strategies, in tandem with the isomorphism relation, gives $\mathsf {M{\hbox {{-}}}Res}$ access to resolution steps that are disallowed in reductionless $\mathsf {LD{\hbox {{-}}}Q{\hbox {{-}}}Res}$.

#### Example 18

Consider the following $\mathsf {M{\hbox {{-}}}Res}$ refutation of the QBF with prefix $\exists x \forall u \exists t$ and clauses $\{x , u , t\}$, $\{{\bar{x}} , {\bar{u}}, t \}$, $\{x, u, {\bar{t}}\}$ and $\{{\bar{x}} , {\bar{u}} , {\bar{t}} \}$.

**Table:** 

Line	Rule	$C_i$	$M_i$	Query
$L_1$	axiom	$\{x , t\}$	$1 \mapsto {\bar{u}}$	
$L_2$	axiom	$\{{\bar{x}}, t\}$	$2\mapsto u$	
$L_3$	$\hbox { res}(L_1,L_2,x)$	$\{t\}$	$\hbox { merge}(M_1,M_2,3,x)$	$3 \mapsto (x,1,2)$
$L_4$	axiom	$\{x,{\bar{t}}\}$	$4 \mapsto {\bar{u}}$	
$L_5$	axiom	$\{{\bar{x}}, {\bar{t}}\}$	$5 \mapsto u$	
$L_6$	$\hbox { res}(L_4,L_5,x)$	$\{{\bar{t}}\}$	$\hbox { merge}(M_4,M_5,6,x)$	$6 \mapsto (x,4,5)$
$L_7$	$\hbox { res}(L_3,L_6,t)$	$\{\}$	$\hbox { select}(M_3,M_6) = M_3$	

**Fig. 5:**
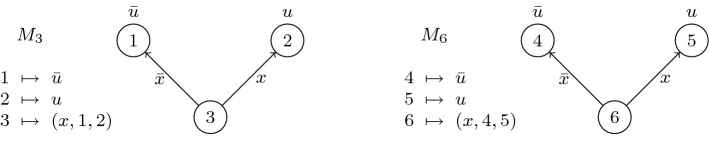
Functions and branching programs for merge maps $M_3$ and $M_6$ from Example 18

As shown in Fig. 5, $M_3$ and $M_6$ are isomorphic, so $\hbox { select}(M_3,M_6)$ is defined and equal to $M_3$. For this reason, the resolution of antecedents $L_3$ and $L_6$ into $L_7$ is allowed, and the final merge map $M_7$ is simply a copy of $M_3$. The analogous resolution would be disallowed in reductionless $\mathsf {LD{\hbox {{-}}}Q{\hbox {{-}}}Res}$ because the pivot *t* is right of *u*, and the non-constant merge maps $M_3$ and $M_6$ would appear as merged literals $\{u,{\bar{u}}\}$ in the antecedent clauses.

We conclude this subsection by showing that the number of lines really is the correct size measure for Merge Resolution. The justification lies in the fact that the domain of the merge map at line *i* is a subset of [*i*].

#### Proposition 19

Let $(C_1,\{M^u_1 : u \in U\}), \dots , (C_k,\{M^u_k : u \in U\})$ be an $\mathsf {M{\hbox {{-}}}Res}$ refutation of ${\mathcal {Q}}\cdot {\phi }$. For each $u \in U$, $M^u_1, \dots , M^u_n$ are pairwise consistent merge maps for *u* over $L_\mathcal {Q}(u)$ with $\max (\hbox { dom}(M^u_i)) \le i$ for each $i \in [n]$.

#### Proof

The claim follows straightforwardly from three observations: (1) each $M^u_i$ introduces at most one node, which is labelled *i*; (2) if $L_i$ is an axiom, then each $M^u_i$ is a merge map over $L_\mathcal {Q}(u)$; (3) the merge operation is only applied when $x \in L_\mathcal {Q}(u)$. $\square$

### Soundness and completeness of $\mathsf {M{\hbox {{-}}}Res}$

The soundness of $\mathsf {M{\hbox {{-}}}Res}$ comes down to the fact that the merge maps at a given line form a partial strategy for the input QBF, in the technical sense of [54]. This means that any total existential assignment that falsifies the clause $C_i$ will falsify the matrix when extended by the output of the merge maps $M^u_i$. Our proof of soundness is an induction on the proof structure with exactly this invariant. At the conclusion, all existential assignments falsify the empty clause $C_k$, and hence the $M^u_k$ compute a countermodel. A trivial corollary, then, is that $\mathsf {M{\hbox {{-}}}Res}$ has linear strategy extraction in merge maps. Our formal proof of soundness is preceded by a preliminary proposition.

#### Proposition 20

Let $M_1$ and $M_2$ be consistent merge maps for *u* over *X* with domains $N_1$ and $N_2$, let $n > \max (N_1 \cup N_2)$ be a natural number, let $x \in X$ and let $\alpha \in \langle X \rangle$. Further, let $h_1, h_2$ and *h* be the functions computed by $M_1$, $M_2$ and $\hbox { merge}(M_1,M_2,x,n)$. Then $h(\alpha ) = h_1(\alpha )$ if ${\bar{x}} \in \alpha$, and $h(\alpha ) = h_2(\alpha )$ if $x \in \alpha$.

#### Proof

Let $M := \hbox { merge}(M_1,M_2,n,x)$, and suppose that ${\bar{x}} \in \alpha$. By Definition 14, $M(n) = (x,\max (N_1),\max (N_2))$ and $M(i) = M_1(i)$ for each $i \in N_1$. Hence, the computation of $h(\alpha )$ from the second iteration of the while loop is identical to the computation of $h_1(\alpha )$ from the first iteration, and it follows that $h(\alpha ) = h_1(\alpha )$. Suppose instead that $x \in \alpha$. By Definition 14, $M(i) = M_2(i)$ for each $i \in N_2 {\setminus } N_1$; by Definition 11, $M_1(i) = M_2(i)$ for each $i \in N_1 \cap N_2$. Then $M(i) = M_2(i)$ for each $i \in N_2$, and the proposition follows as in first case. $\square$

#### Lemma 21

Let $(\emptyset ,\{M^u:u \in U\})$ be the conclusion of an $\mathsf {M{\hbox {{-}}}Res}$ refutation of a QBF $\varPhi$. Then the functions computed by $\{M^u:u \in U\}$ form a countermodel for $\varPhi$.

#### Proof

Let $\pi := L_1,\dots ,L_k$ be an $\mathsf {M{\hbox {{-}}}Res}$ refutation of a QBF $\varPhi := {\mathcal {Q}}\cdot {\phi }$, where each $L_i = (C_i,\{M^u_i:u \in U\})$. Further, for each $i \in [k]$,

- let $\alpha _i := \{{\bar{l}} : l \in C_i\}$ be the smallest assignment falsifying $C_i$,
- let $A_i := \{\alpha \in \langle X \rangle : C_i \cap \alpha = \emptyset \}$ be all assignments to *X* consistent with $\alpha _i$,
- for each $u \in U$, let $h^u_i$ be the function computed by $M^u_i$,
- for each $\alpha \in A_i$, let $l^u_i(\alpha ) := h^u_i(\hbox { proj}(\alpha ,L_\mathcal {Q}(u)))$ and $h_i(\alpha ) := \{l^u_i(\alpha ) : u \in U\} {\setminus } \{*\}$.

(Note that Proposition 19 guarantees that each $h^u_i$ is defined.) By induction on $i \in [k]$, we show, for each $\alpha \in A_i$, that the restriction of $\phi$ by $\alpha \cup h_i(\alpha )$ contains the empty clause. Since $\alpha _k$ is the empty assignment, we have $A_k = \langle X \rangle$. We therefore prove the lemma at the final step $i = k$, as we show that $\{h^u_k : u \in U\}$ is a countermodel for $\varPhi$.

For the base case $i = 1$, let $\alpha \in A_1$. As $L_1$ is introduced as an axiom, there exists a clause $C \in \phi$ such that $C_1$ is the existential subclause of *C*, and each $M^u_1$ is the merge map from $\{i\}$ mapping *i* to the falsifying *u*-literal for *C*. Hence, for each $u \in U$, $l^u_1(\alpha )$ is the falsifying *u*-literal for *C*, so $C[\alpha \cup h_1(\alpha )] = \emptyset$.

For the inductive step, let $i \ge 2$ and let $\alpha \in A_i$. The case where $L_i$ is introduced as an axiom is identical to the base case, so we assume that $L_i$ was derived by resolution. Then there exist integers $a,b < i$ and an existential pivot $x \in X$ such that $C_i = \hbox { res}(C_a,C_b,x)$, and each $u \in U$ satisfies either (i) $M^u_i = \hbox { select}(M^u_a, M^u_b)$, or (ii) $x \in L_\mathcal {Q}(u)$, and $M^u_i = \hbox { merge}(M^u_a,M^u_b,i,x)$. Now, suppose on the one hand that ${\bar{x}} \in \alpha$, and let $u \in U$. If *u* satisfies (i) and $M^u_a$ is non-trivial, then $l^u_i(\alpha ) = l^u_a(\alpha )$, and if *u* satisfies (ii) then $l^u_i(\alpha ) = l^u_a(\alpha )$ by Proposition 20. It follows that $l^u_i \ne l^u_a$ only if $l^u_a = *$, and hence $h_a(\alpha ) \subseteq h_i(\alpha )$. Since $C_a \cup \{x\} \subseteq C_i$, we have $\alpha \in A_a$, so the restriction of $\phi$ by $\alpha \cup h_i(\alpha )$ contains the empty clause by the inductive hypothesis. Supposing, on the other hand, that $x \in \alpha$, a similar argument shows that $h_b(\alpha ) \subseteq h_i(\alpha )$. Note that, in this case, if *u* satisfies (i) and $M^u_b$ is non-trivial, then $M^u_a \simeq M^u_b$ and $l^u_i = l^u_a = l^u_b$ by Proposition 10. $\square$

We show the completeness of $\mathsf {M{\hbox {{-}}}Res}$ via the *p*-simulation of reductionless $\mathsf {LD{\hbox {{-}}}Q{\hbox {{-}}}Res}$. The simulation copies precisely the structure of the reductionless $\mathsf {LD{\hbox {{-}}}Q{\hbox {{-}}}Res}$ refutation, while replacing merged literals by merge maps in the natural way.

#### Theorem 22

$$\mathsf {M{\hbox {{-}}}Res}$$ *p*-simulates reductionless $$\mathsf {LD{\hbox {{-}}}Q{\hbox {{-}}}Res}$$.

#### Proof

Let $\varPhi := {\mathcal {Q}}\cdot {\phi }$ be a QBF with existential variables *X* and universal variables *Y*, and let $\pi := C_1, \dots , C_k$ be a reductionless $\mathsf {LD{\hbox {{-}}}Q{\hbox {{-}}}Res}$ refutation of $\varPhi$. We define a sequence $\pi ^\prime := L_1, \dots ,L_n$, in which each $L_i := (C^\prime _i,\{M^u_i : u \in U\})$, and prove that it is an $\mathsf {M{\hbox {{-}}}Res}$ refutation of $\varPhi$.

For each $i \in [k]$, we define $C^\prime _i$ to be the existential subclause of $C_i$. For each $u \in U$, the merge maps are defined recursively as follows: If $C_i$ is an axiom, $M^u_i$ is defined as the merge map over $L_\mathcal {Q}(u)$ with domain $\{i\}$ mapping *i* to the falsifying *u*-literal for $C_i$ (note that this covers the definition of $M^u_1$). If $C_i$ is derived by resolution, say $C_i = \hbox { res}(C_a,C_b,x)$ with $a,b < i$, then

$$\begin{aligned} M^u_i := {\left\{ \begin{array}{ll} \hbox { select}(M^u_a,M^u_b)\,,&{}\text{ if } \hbox { select}(M^u_a,M^u_b) \text{ is } \text{ defined }\,,\\ \hbox { merge}(M^u_a,M^u_b,i,x)\,,&{}\text{ otherwise }\,.\\ \end{array}\right. } \end{aligned}$$

Now, by induction on $i \in [k]$, we prove that, for each $u \in U$,

- if $\{u,{\bar{u}}\} \nsubseteq C_i$, then $M^u_i$ is isomorphic to $1 \mapsto l$, where *l* is the falsifying *u*-literal for $C_i$,
- $L_i$ can be derived from previous lines in $\pi ^\prime$ using an $\mathsf {M{\hbox {{-}}}Res}$ rule.

Both are established trivially when $C_i$ is an axiom; hence it remains to show the inductive step in the case where $C_i$ was derived by resolution. In this case $C_i = \hbox { res}(C_a,C_b,x)$ for some $a,b < i$ and some $x \in X$.

- Suppose that $\{u,{\bar{u}}\} \nsubseteq C_i$, and let $l_i,l_a,l_b$ be the falsifying *u*-literals for $C_i,C_a,C_b$. By definition of resolution, either (1) $l_i = l_a = l_b$, or (2) exactly one of $l_a,l_b$ is trivial ($l_b$, say), the other is equal to $l_i$. In the former case, $M^u_a$ and $M^u_b$ are both isomorphic to $1 \mapsto l_i$, by the inductive hypothesis; in the latter case, $M^u_a$ is isomorphic to $1 \mapsto l_i$ and $M^u_b$ is trivial. Either way we get $M^u_i = \hbox { select}(M^u_a,M^u_b) = M^u_a$, and the inductive step follows.
- By Proposition 19, for each $u \in U$, $M^u_a$ and $M^u_b$ are consistent merge maps for *u* over $L_\mathcal {Q}(u)$, so $\hbox { merge}(M^u_a,M^u_b,i,x)$ is defined for any case. Hence, if we can show that $\hbox { select}(M^u_a,M^u_b)$ is defined whenever $u <_\mathcal {Q}x$, then it is clear that $L_i$ can be derived by resolution from $L_a$ and $L_b$. To that end, let *u* be left of *x*. If $\{u,{\bar{u}}\} \nsubseteq C_i$, then $\hbox { select}(M^u_a,M^u_b)$ is defined by (a). Otherwise, we must have $u \notin \hbox { vars}(C_a) \cap \hbox { vars}(C_b)$, so the falsifying *u*-literal for one of $C_a$ and $C_b$ is $*$ By the inductive hypothesis, one of $M^u_a$ and $M^u_b$ is trivial, and $\hbox { select}(M^u_a,M^u_b)$ is defined.

This completes the induction. Since $C_n$ contains only universal variables, $C^\prime _k$ is the empty clause, and $\pi ^\prime$ is a refutation. $\square$

With soundness and completeness established by Lemma 21 and Theorem 22, it remains to show that $\mathsf {M{\hbox {{-}}}Res}$ refutations can be checked in polynomial time. This is easy to see, since the isomorphism and consistency relations are computable efficiently.

#### Theorem 23

$\mathsf {M{\hbox {{-}}}Res}$ is a QBF proof system.

## Proof complexity: merge resolution versus reductionless $\mathsf {LD{\hbox {{-}}}Q{\hbox {{-}}}Res}$

In this section we exponentially separate $\mathsf {M{\hbox {{-}}}Res}$ from reductionless $\mathsf {LD{\hbox {{-}}}Q{\hbox {{-}}}Res}$. The separating formulas are a kind of ‘squaring’ of the equality formulas from Definition 2.

### Definition 24

(*squared equality formulas*) The *squared equality family* is the QBF family whose $n^{\hbox {{th}}}$ instance  has the prefix

$$\begin{aligned} \mathcal {Q}(n) := \exists \{x_1,y_1, \dots , x_n,y_n\} \forall \{u_1,v_1, \dots , u_n,v_n\} \exists \{t_{i,j} : i,j \in [n]\}, \end{aligned}$$

and the matrix  consisting of the clauses

The only winning strategy for the universal player is to set $u_i = x_i$ and $v_j = y_j$ for each $i,j \in [n]$. At the final block, the existential player is faced with the full set of $\{t_{i,j}\}$ unit clauses, and to satisfy all of them is to falsify the square clause $\{{\bar{t}}_{i,j} : i,j \in [n]\}$. No other strategy can be winning, as it would fail to produce all $n^2$ unit clauses.

###  lower bound for reductionless $\mathsf {LD{\hbox {{-}}}Q{\hbox {{-}}}Res}$

We first give a formal definition of a refutation *path*; that is, a sequence of consecutive resolvents beginning with an axiom and ending at the conclusion.

#### Definition 25

(*path*) Let $\pi$ be a reductionless $\mathsf {LD{\hbox {{-}}}Q{\hbox {{-}}}Res}$ refutation. A *path* from a clause *C* in $\pi$ is a subsequence $C_1, \dots , C_k$ of $\pi$ in which:

- $C = C_1$ is an axiom of $\pi$;
- $C_k$ is the conclusion of $\pi$;
- for each $i \in [k-1]$, there exists a literal $p_i$ and a clause $R_i$ occurring before $C_{i+1}$ in $\pi$ such that $C_{i+1} = \hbox { res}(C_i,R_i,p_i)$.

The lower-bound proof is based upon two facts: (1) every total existential assignment corresponds to a path, all of whose clauses are consistent with the assignment (Lemma 26); (2) every path from the square clause contains a ‘wide’ clause containing either all the $x_i$ or all the $y_j$ variables (Lemma 27). It is then possible to deduce the existence of exponentially many wide clauses, i.e. by considering the set of assignments for which each $x_i = y_i$ and each $t_{i,j} = 0$, all of whose corresponding paths begin at the square clause (proof of Theorem 28).

#### Lemma 26

Let $\pi$ be a reductionless $\mathsf {LD{\hbox {{-}}}Q{\hbox {{-}}}Res}$ refutation of a QBF $\varPhi$, and let *A* be a clause with $\hbox { vars}(A) = \hbox { vars}_\exists (\varPhi )$. Then there exists a path in $\pi$ in which no existential literal outside of *A* occurs.

#### Proof

We describe a procedure that constructs a sequence $P := C_k,\dots ,C_1$ of clauses in reverse order as follows: To begin with, let the ‘current clause’ $C_1$ be the conclusion of $\pi$. As soon as the current clause $C_i$ is in an axiom, the procedure terminates. Whenever necessary, obtain $C_{i+1}$ as follows: find clauses $R_1$ and $R_2$ occurring before $C_i$ in $\pi$ and a literal $p \in A$ such that $C_i$ is $\hbox { res}(R_1,R_2,p)$, and set $C_{i+1} := R_1$ as the current clause. *P* is clearly a path in $\pi$ by construction. By induction one shows that the existential subclause of $C_i$ is a subset of *A*, for each $i \in [k]$: The base case $i=1$ holds trivially since there are no existential literals in the conclusion $C_1$ of $\pi$. For the inductive step, observe that $C_{i+1} = C^\prime \cup \{p\}$, for some subset $C^\prime \subseteq C_i$ and literal $p \in A$. $\square$

The second lemma is more technical, and its proof more involved. The proof works directly on the definition of path, the rules of reductionless $\mathsf {LD{\hbox {{-}}}Q{\hbox {{-}}}Res}$, and the syntax of the squared equality formulas, to show the existence of the wide clause.

#### Lemma 27

Let $n \ge 2$, and let $\pi$ be a reductionless $\mathsf {LD{\hbox {{-}}}Q{\hbox {{-}}}Res}$ refutation of . On each path from $\{{\bar{t}}_{i,j} : i,j \in [n]\}$ in $\pi$, there occurs a clause *C* for which either $\{x_1, \dots , x_n\} \subseteq \hbox { vars}(C)$ or $\{y_1, \dots , y_n\} \subseteq \hbox { vars}(C)$.

#### Proof

Put $X := \{x_1, \dots , x_n\}$ and $Y := \{y_1, \dots , y_n\}$. Call a clause *R* in $\pi$ a *p-resolvent* if there exist earlier clauses $R_1$ and $R_2$ such that $R = \hbox { res}(R_1,R_2,p)$.

Let $P := C_1, \dots , C_k$ be a path from $\{{\bar{t}}_{i,j} : i,j \in [n]\}$ in $\pi$. With each $C_l$ we associate an $n \times n$ matrix $M_l$ in which $M_l[i,j] := 1$ if ${\bar{t}}_{i,j} \in C_i$ and $M_l[i,j] := 0$ otherwise. Let *l* be the least integer such that $M_l$ has either a 0 in each row or a 0 in each column. Note that $l \ge 2$ since $M_1$ has no zeros.

We prove the lemma by showing that either $X \subseteq \hbox { vars}(C_l)$ or $Y \subseteq \hbox { vars}(C_l)$ must hold.

Suppose that $M_l$ has a 0 in each row. We make use of the following claims, which hold for all $i,j \in [n]$:

1. for each clause *C* on *P*, if ${\bar{t}}_{i,j} \in C$ then $\{u_i, {\bar{u}}_i\} \nsubseteq C$;
2. each $x_i$-resolvent in $\pi$ contains $\{u_i, {\bar{u}}_i\}$ as a subset;
3. for each $t_{i,j}$-resolvent *R* in $\pi$, if $x_i \notin \hbox { vars}(R)$ then $\{u_i, {\bar{u}}_i\} \subseteq R$.

We proceed to show that every row in $M_l$ also has at least one 1. To see this, suppose on the contrary that $M_l$ contains a full 0 row *r* (this implies that $l \ge 2$, and hence that $M_{l-1}$ exists). Note that by definition of resolution there can be at most one element that changes from 1 in $M_{l-1}$ to 0 in $M_l$. Since $M_{l-1}$ does not have a 0 in every column, it does not contain a full zero row. Hence it must be the case that the unique element that went from 1 in $M_{l-1}$ to 0 in $M_{l}$ is in row *r*. Since $n \ge 2$, we deduce that $M_{l-1}$ has a 0 in each row, contradicting the minimality of *l*.

Let $i \in [n]$. Since the $i{{\mathrm{th}}}$ row in $M_l$ contains a 1, there is some $j \in [n]$ for which ${\bar{t}}_{i,j} \in C_l$. From claim (1) it follows that $\{u_i, {\bar{u}}_i\} \nsubseteq C_l$. Moreover, as universal literals accumulate along the path, this means that $\{u_i, {\bar{u}}_i\} \nsubseteq C_m$ for each $m \le l$. Since the $i{{\mathrm{th}}}$ row in $M_l$ contains a 0, there exists $j^\prime \in [n]$ such that ${\bar{t}}_{i,j^\prime } \notin C_l$. As ${\bar{t}}_{i,j^\prime } \in C_1$, there must be a $t_{i,j^\prime }$-resolvent $C_{l^\prime }$ on *P* with $l^\prime \le l$. Then we have $x_i \in \hbox { vars}(C_{l^\prime })$ by claim (3). Also, for each $m \le l$, $C_m$ is not an $x_i$-resolvent by claim (2). It follows that $x_i \in \hbox { vars}(C_l)$. Since $i \in [n]$ was chosen arbitrarily, we have $X \subseteq \hbox { vars}(C_l)$.

Suppose on the other hand that $M_l$ does not contain a 0 in each row. Then $M_l$ contains a 0 in each column. A symmetrical argument, with analogous claims involving the $v_j,y_j$ variables, then shows that $Y \subseteq \hbox { vars}(C_l)$.

It remains to prove the three claims.

1. Observe that each clause in $\pi$ containing the positive literal $t_{i,j}$ also contains the variable $u_i$ (this holds for every axiom and universal literals are never removed). Let *C* be a clause on the path *P* for which ${\bar{t}}_{i,j} \in C$, and, for the sake of contradiction, suppose that $\{u_i, {\bar{u}}_i\} \subseteq C$. Since $u_i <_{\mathcal {Q}(n)} t_{i,j}$, there cannot be $t_{i,j}$-resolvent on *P* following *C*, as such a resolution step is explicitly forbidden in the rules of reductionless $\mathsf {LD{\hbox {{-}}}Q{\hbox {{-}}}Res}$. This means that ${\bar{t}}_{i,j}$ occurs in $C_k$, the final clause of *P*. This is a contradiction, since $C_k$ is the conclusion of $\pi$, which contains no existential literals. Therefore $\{u_i,\bar{u_i}\} \nsubseteq C$.
2. Observe that each clause in $\pi$ containing $x_i$ (resp. ${\bar{x}}_i$) also contains $u_i$ (resp. ${\bar{u}}_i$) (again, this holds for every axiom and universal literals are never removed). Let *R* be an $x_i$-resolvent of $R_1$ and $R_2$ in $\pi$. Since $x_i \in R_1$ and ${\bar{x}}_i \in R_2$, we must have $u_i \in R_1$ and ${\bar{u}}_i \in R_2$. It follows immediately that $\{u_i, {\bar{u}}_i\} \subseteq R$.
3. Observe that each axiom in $\pi$ containing the positive literal $t_{i,j}$ contains variable $x_i$. Hence, any clause in $\pi$ that contains literal $t_{i,j}$ but not variable $x_i$ must appear after an $x_i$-resolvent on some path, and therefore contains $\{u_i,\bar{u_i}\}$ by Claim (2). Now, let *R* be a $t_{i,j}$-resolvent of $R_1$ and $R_2$ in $\pi$. Suppose that $x_i \notin \hbox { vars}(R)$, which implies that $x_i \notin \hbox { vars}(R_1)$. Since $t_{i,j} \in R_1$, we have $\{u_i, {\bar{u}}_i\} \subseteq R_1$, and it follows that $\{u_i, {\bar{u}}_i\} \subseteq R$.

$$\square $$

It remains to prove the lower bound formally from the preceding lemmata.

#### Theorem 28

The squared equality family requires exponential-size reductionless $\mathsf {LD{\hbox {{-}}}Q{\hbox {{-}}}Res}$ refutations.

#### Proof

Let $n \in {\mathbb {N}}$, and let $\pi$ be a reductionless $\mathsf {LD{\hbox {{-}}}Q{\hbox {{-}}}Res}$ refutation of . We show that $|\pi | \ge 2^{n-1}$. The size bound is trivially true for $n=1$, so we assume $n \ge 2$. Put $X := \{x_1, \dots , x_n\}$ and $Y := \{y_1, \dots , y_n\}$, and let $L := \{{\bar{t}}_{i,j}:i,j \in [n]\}$ be the long clause from . We call a non-tautological clause *S* *symmetrical* iff $\hbox { vars}(S) = X \cup Y$ and $x_i \in S \Leftrightarrow y_i \in S$ for each $i \in [n]$. (A symmetrical clause represents a total assignment to $X \cup Y$). Note that there are $2^n$ distinct symmetrical clauses.

By Lemma 26, for each symmetrical clause *S*, there exists a path $P_S$ in $\pi$ in which all existential literals are contained in $S \cup L$. Moreover, each $P_S$ begins at clause *L*, since every other clause in $\hbox {{eq}}^2(n)$ contains some positive $t_{i,j}$ literal that does not occur in $S \cup L$. By Lemma 27, on each path *P* from *L* in $\pi$ there exists a clause *C* for which either $X \subseteq \hbox { vars}(C)$ or $Y \subseteq \hbox { vars}(C)$. It follows that we can define a function *f* that maps each symmetrical assignment *S* to a clause *f*(*S*) in $\pi$ for which either $\hbox { proj}(S,X) \subseteq f(S)$ or $\hbox { proj}(S,Y) \subseteq f(S)$. Moreover, since distinct symmetrical clauses $S_1$ and $S_2$ satisfy $\hbox { proj}(S_1,X) \ne \hbox { proj}(S_2,X)$ and $\hbox { proj}(S_1,Y) \ne \hbox { proj}(S_2,Y)$, each *f*(*S*) is the image of at most two distinct symmetrical clauses. Hence, $\pi$ contains at least $2^{n-1}$ clauses. $\square$

Close inspection of the lower-bound proof reveals that particular resolution steps are blocked due to the appearance of merged literals in the antecedents (see the proof of claim (1) of Lemma 27). As we noted in Example 18, such steps remain blocked even if both merged literals implicitly represent the same (non-constant) function, in which case the resolution step is actually perfectly sound. As we will see, the $\mathsf {M{\hbox {{-}}}Res}$ upper-bound construction makes crucial use of the isomorphism of non-constant merge maps.

### Short $\mathsf {M{\hbox {{-}}}Res}$ refutations of 

Here we construct short $\mathsf {M{\hbox {{-}}}Res}$ refutations of the squared equality formulas. The approach is as follows. First, for each $i,j \in [n]$, obtain a line $(\{t_{i,j}\}, M_{i,j})$ by resolving the axioms for the four clauses in $\hbox {{eq}}(n)^2$ that contain $\{t_{i,j}\}$. By the natural application of the $\hbox { merge}$ and $\hbox { select}$ operations, one obtains merge maps $M_{i,j}$ in which the merge map for $u_i$ outputs $x_i$ with a single query, the merge map for $v_j$ outputs $y_j$ with a single query, and all other maps are trivial. Notice that all the non-trivial merge maps for a given universal variable are isomorphic, so these $n^2$ unit clauses can all be resolved against the square clause, utilising the select operation. It is precisely this final step which is unavailable in reductionless $\mathsf {LD{\hbox {{-}}}Q{\hbox {{-}}}Res}$.

#### Theorem 29

The squared equality family has $O(n^2)$-size $\mathsf {M{\hbox {{-}}}Res}$ refutations.

#### Proof

Let $n \in {\mathbb {N}}$. We construct a refutation in two stages. In the first stage we explicitly construct an $\mathsf {M{\hbox {{-}}}Res}$ derivation $\pi := L_1, \dots , L_k$ from , where $k = 2n^2$. In the second stage, we show that $\pi$ can be extended to a refutation with a further $n^2 + 1$ lines.

**Stage one.** For each $h,i,j \in {\mathbb {N}}$ we let $\delta (h,i,j) := (h-1)n^2 + (i-1)n + j$ and use *L*(*h*, *i*, *j*) as an alias for $L_{\delta (h,i,j)}$. Similarly, we let *C*(*h*, *i*, *j*) be the clause, *U*(*h*, *i*, *j*) be the merge map for $u_i$, and *V*(*h*, *i*, *j*) be the merge map for $v_j$ appearing on line *L*(*h*, *i*, *j*). These *U*(*h*, *i*, *j*) and *V*(*h*, *i*, *j*) are the only merge maps in $\pi$ we define explicitly; we consider all others to be defined implicitly as the appropriate trivial merge map.

Letting $i,j \in [n]$, we define the first $4n^2$ lines withand observe that each of these lines can be introduced as an axiom.

The next $2n^2$ lines are the result of the natural resolutions over $y_j$. For each $i,j \in [n]$ we defineEach line *L*(4, *i*, *j*) can be derived by resolution from *L*(0, *i*, *j*) and *L*(2, *i*, *j*); to see this, note that *U*(0, *i*, *j*) is clearly isomorphic to *U*(2, *i*, *j*) and *V*(0, *i*, *j*) is trivially consistent with *V*(2, *i*, *j*) (their domains are disjoint), therefore $U(4,i,j) = \hbox { select}(U(0,i,j),U(2,i,j))$ and

$$\begin{aligned} V(4,i,j) = \hbox { merge}(V(0,i,j),V(2,i,j),\delta (4,i,j),y_j)\,. \end{aligned}$$

A similar argument shows each that *L*(5, *i*, *j*) can be derived by resolution from *L*(1, *i*, *j*) and *L*(3, *i*, *j*).

The final $n^2$ lines are the result of the natural resolutions over $x_i$. For each $i,j \in [n]$ we defineIt is easy to see that each *L*(6, *i*, *j*) can be derived by resolution from *L*(4, *i*, *j*) and *L*(5, *i*, *j*), since *V*(4, *i*, *j*) is clearly isomorphic to *V*(5, *i*, *j*) (an isomorphism is $l \mapsto l+n^2$) and *U*(0, *i*, *j*) is trivially consistent with *U*(1, *i*, *j*) (disjoint domains).

**Stage two.** We now show how $\pi$ can be extended to a refutation. Let $L_6 := \{L(6,i,j) : i,j \in [n]\}$ denote the final $n^2$ lines of $\pi$, in each of which appears some unit clause $\{t_{i,j}\}$. We observe that, for each $a,b,i \in [n]$, *U*(6, *i*, *a*) is isomorphic to *U*(6, *i*, *b*) (an isomorphism is $l \mapsto l + b - a$); that is, amongst the lines $L_6$, the non-trivial merge maps for $u_i$ are pairwise isomorphic. Similarly, for each $j \in [n]$, the non-trivial merge maps for $v_j$ appearing in $L_6$ are pairwise isomorphic.

Now, a line *T*, consisting of the clause $\{{\bar{t}}_{i,j} : i,j \in [n]\}$ and a full set of trivial merge maps, can be introduced as an $\mathsf {M{\hbox {{-}}}Res}$ axiom in a derivation from . From *T* and $L_6$, in a further $n^2$ steps we obtain a refutation by successively resolving each line in $L_6$ against *T*, removing a literal ${\bar{t}}_{i,j}$ each time. All such resolution steps are valid, since the merge map for $u_i$ ($v_j$) in any line can be defined as $\hbox { select}(M_a,M_b)$, where $M_a$ and $M_b$ are the merge maps for $u_i$ appearing in the antecedent lines. The isomorphism of non-trivial merge maps for $u_i$ ($v_j$) is preserved, and ensures that $\hbox { select}(M_a,M_b)$ is defined. $\square$

The separation follows immediately from Theorems 28 and 29.

#### Theorem 30

$\mathsf {LD{\hbox {{-}}}Q{\hbox {{-}}}Res}$ does not *p*-simulate $\mathsf {M{\hbox {{-}}}Res}$ on QBF.

## Overview of DQBF

In this section, we provide an overview of DQBF, which will help to explain how Merge Resolution is best extended to a DQBF proof system (in Sect. 7).

### S-form versus H-form

A DQBF can be written in one of two forms: Skolem-form (S-form) and Herbrand-form (H-form) [2]. To date, most of the DQBF literature has focused on S-form (whether in computational complexity [1, 16], proof complexity [8, 50], and solving [27, 29, 55, 57, 58]), whereas relatively little has been written about H-form [2]. The DQBF solver presented in [25] uses H-form DQBF to facilitate a reduction to QBF. Otherwise, as far as we are aware, existing DQBF solvers use S-form exclusively [52].

We will recall S-form and H-form DQBFs, their semantics, and the transformation operation that relates them.

An *S-form dependency quantified Boolean formula* (DQBF) is a formula of the form

1 $$\begin{aligned} \varPhi := {\forall u_1 \cdots \forall u_m \exists x_1(S_1) \cdots \exists x_n(S_n)}\cdot {\phi }\,, \end{aligned}$$

in which $\phi$ is a CNF, and each $S_i$ is a subset of the universally quantified variables $\{u_1, \dots ,u_m\}$. S-form DQBF generalises QBF, since the quantifier prefix has a more general specification that allows variable dependencies for the existentials to be written explicitly in the sets $S_i$. QBF is the fragment of S-form DQBF for which the dependency sets are nested subsets, i.e. $S_1 \subseteq S_2 \subseteq \cdots \subseteq S_n$.

An S-form DQBF is true if and only if it has a Skolem-function model. A Skolem-function model *g* for $\varPhi$ is a set $\{g_i : i \in [n]\}$ of functions

$$\begin{aligned} g_i : \langle S_i \rangle \rightarrow \{x_i, {\bar{x}}_i\} \end{aligned}$$

such that, for each $\alpha \in \langle \{u_1, \dots ,u_m\} \rangle$,

$$\begin{aligned} \alpha \cup \{g_i({\alpha }{\upharpoonright }_{S_i}) : i \in [n]\} \text{ satisfies } \phi \,. \end{aligned}$$

An *H-form* DQBF is the obvious dual to S-form, namely a formula of the form

$$\begin{aligned} \varPsi := {\exists x_1 \cdots \exists x_n \forall u_1(H_1) \cdots \forall u_m(H_m)}\cdot {\phi }\,, \end{aligned}$$

in which $\phi$ is a CNF, and each $H_i$ is a subset of the existentially quantified variables $\{x_1, \dots ,x_n\}$. Here the $H_i$ express the variable dependencies for the universals, as opposed to the existentials in S-form.

An H-form DQBF is false if and only if it has an Herbrand-function countermodel, which is dual to a Skolem-function model. An Herbrand-function countermodel *h* for $\varPsi$ is a set $\{h_i : i \in [m]\}$ of functions

$$\begin{aligned} h_i : \langle H_i \rangle \rightarrow \{u_i, {\bar{u}}_i\} \end{aligned}$$

such that, for each $\beta \in \langle \{x_1, \dots ,x_n\} \rangle$,

$$\begin{aligned} \beta \cup \{h_i({\beta }{\upharpoonright }_{H_i}) : i \in [m]\} \text{ falsifies } \phi \,. \end{aligned}$$

The dual definitions of S-form and H-form DQBF seem perfectly natural, and both sets of formulas generalise QBF in an obvious way. Nonetheless, it was shown in [2] that the situation in terms of semantics is already quite complex. To see this, consider the transformation operation *T* defined below. (It is a combination of the negation and complement operators defined in [2]. We find it more convenient here to have a single operation.) This operator is a natural map from S-form onto H-form DQBF and from H-form onto S-form DQBF. The *T*-transform of the S-form DQBF in (1) is the H-form DQBF

$$\begin{aligned} T(\varPhi ) := {\exists x_1 \cdots \exists x_n \forall u_1(H^\prime _1) \cdots \forall u_m(H^\prime _m)}\cdot {\phi }\,, \end{aligned}$$

where $H^\prime _i := \{x_j : u_i \notin S_j\}$. Intuitively, in the transformed H-form, a universal variable *u* depends on the existentials which did not depend on *u* in the original S-form. The *T*-transform of the H-form DQBF is defined analogously. (In the notation of [2], for any DQBF $\varPhi$, $T(\varPhi ) = {\lnot } {\sim } {\varPhi } = {\sim } {\lnot } {\varPhi }$.)

It is easy to see that for any DQBF $\varPhi$, $T(T(\varPhi )) = \varPhi$.

To see why the *T*-transform is a natural operation, consider what happens to a QBF. Recall that in an S-form QBF, the dependency sets are nested ($S_1 \subseteq S_2 \subseteq \cdots \subseteq S_n$), therefore the dependency sets in the *T*-transform are also nested ($H^\prime _1 \subseteq H^\prime _2 \subseteq \cdots \subseteq H^\prime _m$). In fact, it is not too hard to see that both collections of dependency sets represent the same (linear) QBF prefix. Therefore, the transform of an S-form QBF is just an H-form representation of the same QBF, and this is verified semantically: an S-form QBF has a Skolem-function model (is true) if and only if its transformed H-form does not have an Herbrand-function countermodel (is not false); and it does not have a Skolem-function model if and only if its transform does have an Herbrand-function countermodel. Thus, every QBF $\varPhi$ is logically equivalent to $T(\varPhi )$; the only change made by the transformation is from S-form to H-form and vice versa.

But this is not the case in general for DQBF. The authors of [2] partitioned S-form DQBF into four distinct classes:

(A) those which have a Skolem-function model, but whose transform has no Herbrand-function countermodel.
(B) those which have no Skolem-function model, but whose transform does have an Herbrand-function countermodel.
(C) those which have a Skolem-function model, and whose transform also has an Herbrand-function countermodel.
(D) those which have no Skolem-function model, nor does their transform have an Herbrand-function countermodel.

All QBFs are either type A or B. Type C and D are classes of DQBFs whose semantic properties are markedly different from QBF.

### Expansion versus QCDCL

Given what we know about the semantics of DQBF, we pose the following question: What is the impact of the existence of type C and D DQBFs on the transfer of solving techniques from QBF? We argue that the impact is indeed visible in theoretical models of solving. Moreover, it forms a decent explanation for the results that we have seen there.

**Fig. 6:**
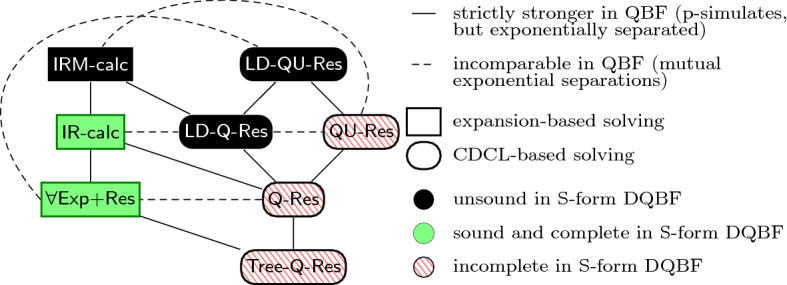
The simulation order of QBF resolution systems and soundness/completeness of their versions lifted to S-form DQBF

Figure 6 (reproduced from [13]) depicts what happens when one attempts to lift various QBF calculi to DQBF. All of these systems are refutational calculi for S-form DQBFs; that is, they prove that an S-form DQBF does not have a Skolem-function model.

The main message of Fig. 6 (and the conclusion of [13]) is that expansion-based systems lift to S-form DQBF whereas CDCL-based systems do not. Q-Resolution, for example, is too weak (it is not complete for S-form DQBF), whereas long-distance Q-Resolution is too strong (it is not sound).

A reasonable explanation for this goes as follows:

*Expansion-based* (D)QBF *calculi prove the non-existence of Skolem functions, whereas CDCL-based* (D)QBF *calculi prove the existence of Herbrand functions.*

Such an explanation could scarcely be sought in the QBF realm, where the non-existence of a Skolem-function model and the existence of an Herbrand-function countermodel are equivalent. One really needs to consider the behaviour of type C and D formulas to understand that these two things are not equivalent for DQBF.

Whereas our statement is not the kind that can be proved as a theorem, there appears good reason to promote it as a credible hypothesis, since it explains the situation depicted in Fig 6.

Expansion-based systems prove that the universal expansion of a (D)QBF (i.e. a propositional formula) is unsatisfiable. Satisfying assignments for the expansion are in one-one correspondence with Skolem-function models, so a proof of unsatisfiability is a proof of the non-existence of Skolem functions. Thus, the expansion systems $\forall {\mathsf {Exp}}{\hbox {{+}}}{\mathsf {Res}}$ and $\mathsf {IR{\hbox {{-}}}calc}$ should lift quite naturally to refutational systems for S-form DQBFs, whose falsity is witnessed by the non-existence of Skolem functions. And indeed, they lift easily to DQBF, as shown in Fig 6 [13].

Moreover, if CDCL-based systems prove the existence of Herbrand functions, we should expect to see difficulties lifting them to S-form DQBF, because the rules of these systems implicitly work on the *T*-transformed formulas, which is an H-form DQBF. We know that there exist type C S-form DQBFs that are true, but whose transform also has Herbrand functions, and type D S-form DQBFs that are false, but whose transform does not have Herbrand functions. In the former case we could expect to refute a true formula (unsoundness), in the latter case we find false formulas that we cannot refute (incompleteness). This is precisely what we see in Fig. 6: $\mathsf {LD{\hbox {{-}}}Q{\hbox {{-}}}Res}$ is unsound for S-form DQBF [13], whereas $\mathsf {Q{\hbox {{-}}}Res}$ is incomplete [2].

Note that $\mathsf {IRM{\hbox {{-}}}calc}$, which is considered an expansion-based system, is also unsound for S-form DQBFs. This is because the system is designed to simulate $\mathsf {LD{\hbox {{-}}}Q{\hbox {{-}}}Res}$, and unfortunately also simulates unsound $\mathsf {LD{\hbox {{-}}}Q{\hbox {{-}}}Res}$ refutations of true S-form DQBFs.

### Switching from S-form to H-form

We suggest, then, that it is worthwhile to investigate further the use of H-form DQBF as an input encoding for CDCL-based DQBF solving. At least for theoretical models, this is yet to be investigated. Here we undertake the first such investigation, and we get some positive results: Merge Resolution lifts naturally to a sound and complete CDCL-based refutational proof system on H-form DQBF.

It should be noted that a resolution system for DQBF called Fork Resolution [50] was shown to be sound and complete for S-form DQBF. The system is based on so-called ‘information forks’, and allows the introduction of fresh variables that delegate the responsibility for fork satisfaction between the original variables. Whereas Fork Resolution is clearly a variant of Q-Resolution, it is not clear whether one should call it a CDCL-based system. Certainly, the associated solver DCAQE [55] belongs to the paradigm of clausal abstraction, rather than conflict-driven clause learning. However, we wish to make it clear that switching to H-form is not the only solution to the issues associated with Fig. 6.

## Extending merge resolution to H-form DQBF

In this section, we show that $\mathsf {M{\hbox {{-}}}Res}$ extends naturally to a proof system for H-form DQBF with the addition of a single weakening rule.

For consistency with the QBF definition, we introduce an equivalent notation for H-form DQBF. We write the quantifier prefix of the H-form DQBF

$$\begin{aligned} \varPhi := {\exists x_1 \cdots \exists x_n \forall u_1(H_1) \cdots \forall u_m(H_m)}\cdot {\phi } \end{aligned}$$

as a triple $\mathcal {Q}:= (X,U,L_\mathcal {Q})$, where:

- $X = \{x_1, \dots ,x_n\}$ is the set of existential variables;
- $U = \{u_1, \dots ,u_m\}$ is the set of universal variables;
- $L_\mathcal {Q}: U \rightarrow \wp (X)$ is the support set function, which maps each $u_i$ to its dependency set $H_i$.

To lift $\mathsf {M{\hbox {{-}}}Res}$ to H-form DQBF, we take $\varPhi$ to be a DQBF in Definition 16 and add an extra case:

(c) **Weakening.** There exists an integer $a < i$ such that $C_i$ is an existential superclause of $C_a$ and, for each $u \in U$, either (i) $M^u_i = M^u_a$, or (ii) $M^u_a$ is trivial and $M^u_i := i \mapsto l$ for some literal $l \in \{u, {\bar{u}}\}$.

By ‘existential superclause’ it is meant that $\hbox { vars}(C_i) \subseteq X$ and $C_a \subseteq C_i$.

Weakening is, in a clear sense, the simplest rule with which one extends $\mathsf {M{\hbox {{-}}}Res}$ to H-form DQBF. Its function is merely to represent exactly the paths of the countermodel on which the canonical completeness construction is based. In general, the countermodel needs to be represented in full since merge maps must be isomorphic in order to apply the select operation. Note that the DQBF analogue of Proposition 19 is proved easily with an additional case for the weakening rule.

### Soundness and completeness

Soundness of $\mathsf {M{\hbox {{-}}}Res}$ for H-form DQBF is proved in the same way as for QBF, i.e. by showing that the concluding merge maps compute a countermodel.

#### Lemma 31

Let $(\emptyset ,\{M^u:u \in U\})$ be the conclusion of an $\mathsf {M{\hbox {{-}}}Res}$ refutation of an H-form DQBF $\varPhi$. Then the functions computed by $\{M^u:u \in U\}$ form a countermodel for $\varPhi$.

#### Proof

We add an additional case to the inductive step in the proof of Lemma 21. Suppose that $L_i$ was derived by weakening. Then there exists an integer $a < i$ such that $C_a \subseteq C_i$ and, for each $u \in U$, either (i) $M^u_i = M^u_a$, or (ii) $M^u_a$ is trivial and $M^u_i := i \mapsto l$ for some literal $l \in \{u, {\bar{u}}\}$. Here $A_i \subseteq A_a$, so $\alpha \in A_a$. For each $u \in U$, if *u* satisfies (i) then $l^u_i(\alpha ) = l^u_a(\alpha )$, and if *u* satisfies (ii) then $l^u_a(\alpha ) = *\notin h_i(\alpha )$. Hence we have $h_a(\alpha ) \subseteq h_i(\alpha )$. It follows that the restriction of $\phi$ by $\alpha \cup h_i(\alpha )$ contains the empty clause by the inductive hypothesis.$\square$

Completeness, on the other hand, cannot be established with an analogue of Theorem 22; DQBF is strictly larger than QBF, and hence simulation of reductionless $\mathsf {LD{\hbox {{-}}}Q{\hbox {{-}}}Res}$ does not guarantee completeness. Our proof rather extends the method by which completeness of reductionless $\mathsf {LD{\hbox {{-}}}Q{\hbox {{-}}}Res}$ was proved in Lemma 5; namely, the construction of a ‘full binary tree’ of resolution steps based on the countermodel, following the prefix order of existential variables.

We give an overview of the construction. Let $\varPhi :={(X,U,L_\mathcal {Q})}\cdot {\phi }$ be a false DQBF with a countermodel *h*. For each $\alpha \in \langle X \rangle$, the assignment $\alpha \cup h(\alpha )$ falsifies some clause $C_\alpha \in \phi$ by definition of countermodel. Now, consider the $\mathsf {M{\hbox {{-}}}Res}$ line whose clause is the largest existential clause falsified by $\alpha$ and whose merge maps are constant functions computing $h(\alpha )$. Each such line can be derived in two $\mathsf {M{\hbox {{-}}}Res}$ steps, by weakening the axiom corresponding to $C_\alpha$. Moreover, the clauses $\{C_\alpha : \alpha \in \langle X \rangle \}$ form the leaves of a full binary tree resolution refutation which can be completed using an arbitrary order of the existential pivots *X*. The merge maps are constructed by merging over the pivot *x* iff $x \in L_\mathcal {Q}(u)$; otherwise the $\hbox { select}$ operation takes the merge map from either antecedent, since the full binary tree structure *guarantees* that they are isomorphic.

As merge maps essentially represent the structure of resolution steps in the subderivation, it is no surprise that the merge maps in our construction also have a full binary tree structure. This structure is captured by the following definition.

#### Definition 32

(*binary tree merge map*) A *binary tree merge map* for a variable *u* over a sequence of variables $x_1, \dots ,x_n$ is a function *M* with domain $[2^{n+1} - 1]$ and rule

$$\begin{aligned} M(i) := {\left\{ \begin{array}{ll} (x_{\lfloor \log i\rfloor +1}, 2i, 2i+1)&{}\text{ if } 1 \le i< 2^n\,,\\ l_i&{}\text{ if } 2^n \le i < 2^{n+1}\,, \end{array}\right. } \end{aligned}$$

where each $$l_i \in \{u, {\bar{u}}\}$$.

At the technical level, we must define existential restrictions for DQBFs and DQBF countermodels. Let $\varPhi := {(X,U,L_\mathcal {Q})}\cdot {\phi }$ be a DQBF with a countermodel *h* and let *l* be a literal with $\hbox { var}(l) = x \in X$. The *restriction* of $\varPhi$ by *l* is $\varPhi [l] := {(X {\setminus } \{x\},U,L^\prime _\mathcal {Q})}\cdot {\phi [l]}$, where $L^\prime _\mathcal {Q}$ maps each $u \in U$ to $L_\mathcal {Q}(u) {\setminus } \{x\}$. The restriction of *h* by *l* is $h[l] := \{h_u[l]: u \in U\}$, where the functions $h_u[l] : \langle L^\prime _\mathcal {Q}(u) \rangle \rightarrow \{u, {\bar{u}}\}$ are defined by $h_u[l](\alpha ) := h_u((\alpha \cup \{l\}){\upharpoonright }_{L_\mathcal {Q}(u)})$.

The construction itself is defined recursively in the completeness proof, combining full binary tree refutations for $\varPhi [x]$ and $\varPhi [{\bar{x}}]$ for some $x \in X$ with a single resolution step. We use the fact that restrictions preserve countermodels in the following sense.

#### Proposition 33

Let *h* be a countermodel for a DQBF $\varPhi := {(X,U,L_\mathcal {Q})}\cdot {\phi }$ and let *l* be a literal with $\hbox { var}(l) \in X$. Then *h*[*l*] is a countermodel for $\varPhi [l]$.

As the final precursor to the completeness proof, we show that a derivation of the negated literal ${\bar{l}}$ and the restricted countermodel *h*[*l*] can be obtained easily from a refutation of the restricted DQBF $\varPhi [l]$

#### Proposition 34

Let $\varPhi :={(X,U,L_\mathcal {Q})}\cdot {\phi }$ be a false DQBF, let *l* be a literal with $\hbox { var}(l) \in X$, and let $(\emptyset ,\{M_u : u \in U\})$ be the conclusion of be an $\mathsf {M{\hbox {{-}}}Res}$ refutation of $\varPhi [l]$. Then there exists an $\mathsf {M{\hbox {{-}}}Res}$ derivation of $(\{{\bar{l}}\},\{M_u : u \in U\})$ from $\varPhi$.

#### Proof

Let $\pi$ be the refutation with the given conclusion. The desired derivation may be obtained from $\pi$ simply by adding the literal $\{{\bar{l}}\}$ to each clause, applying weakening where necessary, and adjusting the indexing of the merge maps to account for the extra weakening steps. $\square$

#### Lemma 35

Every false H-form DQBF has an $\mathsf {M{\hbox {{-}}}Res}$ refutation.

#### Proof

Let $\varPhi :={(X,U,L_\mathcal {Q})}\cdot {\phi }$ be a false DQBF, and let $X := \{x_1, \dots , x_n\}$ where the $x_i$ are pairwise distinct. For any $\mathsf {M{\hbox {{-}}}Res}$ refutation $\pi$ with conclusion $(C_k, \{M^u_k : u \in U\})$, let $\{h_u : u \in U\}$ be the *concluding countermodel* for $\pi$, where the $h_u$ are the functions computed by the *concluding merge maps* $M^u_k$. A merge map for $u \in U$ over $L_\mathcal {Q}(u)$ is said to be *complete* if it is isomorphic to a binary tree merge map for *u* over the sequence

$$\begin{aligned} x_{\sigma (1)}, \dots x_{\sigma (|L_\mathcal {Q}(u)|)}\,, \end{aligned}$$

which enumerates $L_\mathcal {Q}(u)$ in increasing index order; that is, $\sigma : [|L_\mathcal {Q}(u)|] \rightarrow [n]$ is the unique function satisfying $\{x_{\sigma (i)} : i \in [|L_\mathcal {Q}(u)|]\} = L_\mathcal {Q}(u)$ and $i< j \Leftrightarrow \sigma (i) < \sigma (j)$ for each $i,j \in [|L_\mathcal {Q}(u)|]$. By induction on the number *n* of existential variables, we show that, for each countermodel *h* for $\varPhi$, there exists an $\mathsf {M{\hbox {{-}}}Res}$ refutation whose concluding countermodel is *h* and whose concluding merge maps are complete. To that end, let $h := \{h_u : u \in U\}$ be an arbitrary countermodel for $\varPhi$.

For the base case $|X| = 0$, observe that each $h_u$ is a constant function with some singleton codomain $\{l_u\}$. By definition of countermodel, there exists a clause $C \in \phi$ such that $C = \{{\bar{l}}_u : u \in \hbox { vars}(C)\}$. Applying the axiom rule to *C*, one obtains a derivation of the line $(\emptyset ,\{M^u : u \in U\})$ in which $M^u$ computes the constant function $h_u$ if $u \in \hbox { vars}(C)$, and is trivial otherwise. With a single weakening step, each trivial $M^u$ can be swapped for a merge map isomorphic to $1 \mapsto l_u$. Then each $M^u$ is trivially complete and computes the constant function $h_u$.

For the inductive step, let $n \in {\mathbb {N}}$. Combining Propositions 33 and 34 with the inductive hypothesis, we deduce that there exist $\mathsf {M{\hbox {{-}}}Res}$ derivations $\pi$ and $\pi ^\prime$ of the lines $(\{{\bar{x}}_1\}, \{M_u : u \in U\})$ and $(\{x_1\}, \{M^\prime _u : u \in U\})$ from $\varPhi$ in which the $M_u$ and $M^\prime _u$ are complete merge maps computing $h_u[x_1]$ and $h_u[{\bar{x}}_1]$. Assume that the lines of $\pi$ are indexed from 1 to $|\pi |$ and that those of $\pi ^\prime$ are indexed from $|\pi | + 1$ to $|\pi | + |\pi ^\prime |$. For each $u \in U$, the domains of $M_u$ and $M^\prime _u$ are disjoint, so $M_u \bowtie M^\prime _u$. If $x_1 \notin L_\mathcal {Q}(u)$, then $h_u[x_1] = h_u[{\bar{x}}_1]$, and we must have $M_u \simeq M^\prime _u$ since complete merge maps computing the same function must be isomorphic. It follows that the line $(\emptyset , \{M^{\prime \prime }_u : u \in U\})$ can be derived from $\varPhi$, where

$$\begin{aligned} M^{\prime \prime }_u := {\left\{ \begin{array}{ll} \hbox { merge}(M_u,M^\prime _u,|\pi | + |\pi ^\prime | + 1, x_1)&{}\text{ if } x_1 \in L_\mathcal {Q}(u),\\ M_u&{}\text{ if } x_1 \notin L_\mathcal {Q}(u).\\ \end{array}\right. } \end{aligned}$$

It is easy to see that the $M^{\prime \prime }_u$ are complete merge maps computing the $h_u$. $\square$

The weakening rule is clearly polynomial-time checkable. Thus the following is immediate from Lemmata 31 and 35.

#### Theorem 36

$\mathsf {M{\hbox {{-}}}Res}$ is a proof system for H-form DQBF.

It is natural to consider whether the weakening rule is necessary for completeness. This is indeed the case; there exist false H-form DQBFs that cannot be refuted by $\mathsf {M{\hbox {{-}}}Res}$ without weakening.

For example, consider the DQBF $\varPhi := {(X,U,L_\mathcal {Q})}\cdot {\phi }$ in which $X := \{x_1, x_2\}$, $U := \{u_1, u_2\}$, the support set function is given by

$$\begin{aligned} L_\mathcal {Q}(u_1) = \{x_1\}, L_\mathcal {Q}(u_2) = \{x_2\}\,, \end{aligned}$$

and the matrix $\phi$ consists of the clauses

$$\begin{aligned} \{{\bar{x}}_1,{\bar{x}}_2,{\bar{u}}_1,{\bar{u}}_2\}, \{x_1,x_2, u_1, u_2\},\{{\bar{x}}_1,x_2\}, \{x_1,{\bar{x}}_2\}. \end{aligned}$$

It is easy to see that the only countermodel for $\varPhi$ sets $u_1 = x_1$ and $u_2 = x_2$. Note that the functions computing this unique countermodel have ranges $\{{\bar{u}}_1,u_1\}$ and $\{{\bar{u}}_2,u_2\}$

Now, let $\pi$ be a weakening-free $\mathsf {M{\hbox {{-}}}Res}$ derivation from $\varPhi$. We will show that each line in $\pi$ is of one of three types:

A The merge maps compute functions with ranges $R^A_1$ and $R^A_2$, where
  $$\begin{aligned} \{u_1\} \subseteq R^A_1 \subseteq \{*, u_1\} \text{ and } \{u_2\} \subseteq R^A_2 \subseteq \{ *, u_2\}\,; \end{aligned}$$
B The merge maps compute functions with ranges $R^B_1$ and $R^B_2$, where
  $$\begin{aligned} \{{\bar{u}}_1\} \subseteq R^B_1 \subseteq \{*, {\bar{u}}_1\} \text{ and } \{{\bar{u}}_2\} \subseteq R^B_2 \subseteq \{ *, {\bar{u}}_2\}\,; \end{aligned}$$
C The merge maps compute functions with ranges $R^C_1 = R^C_2 =\{*\}$.

From this it follows that $\pi$ is not a refutation, because its concluding merge maps do not compute a countermodel.

The axiom line for the clause $\{{\bar{x}}_1,{\bar{x}}_2,{\bar{u}}_1,{\bar{u}}_2\}$ is type A, the axiom line for the clause $\{x_1,x_2, u_1, u_2\}$ is type B, and the remaining two clauses, which contain no universal literals, are type C. It is easy to see that resolution of a type A line with a type A or C line always yields type A. Similarly, resolution of a type B line with a type B or C line always yields type B. Resolving two type C clauses yields a type C clause. Moreover, type A lines can never be resolved with type B lines; in this case, the merge maps for $u_1$ are non-trivial and non-isomorphic, and similarly for $u_2$, so neither $x_1$ nor $x_2$ is eligible as the pivot variable.

## Conclusions and future work

### What is new in $\mathsf {M{\hbox {{-}}}Res}$?

To the best of our knowledge, $\mathsf {M{\hbox {{-}}}Res}$ is the first ‘long-distance’ proof system for DQBF. Recent work [13] showed that the DQBF version of $\mathsf {LD{\hbox {{-}}}Q{\hbox {{-}}}Res}$ is not sound, so it is natural to ask how $\mathsf {M{\hbox {{-}}}Res}$ fares in comparison. We identify three major differences.

Firstly, $\mathsf {M{\hbox {{-}}}Res}$ works with Herbrand-form DQBFs, whereas the system in [13] was defined for Skolem-form DQBFs (which use support sets for *existential* variables). Merge maps detail precisely how the Herbrand functions are encoded in the resolution structure of long-distance proofs. One could say that such refutations are ‘proving the existence of Herbrand functions’. For QBF, this is of course equivalent to proving the non-existence of Skolem functions, but that does not carry over to DQBF (in a precise technical sense [2]). From this standpoint, it is natural to refute H-form DQBFs by finding the Herbrand functions that certify the falsity of the formula, and this is exactly what $\mathsf {M{\hbox {{-}}}Res}$ achieves. On the other hand, [13] takes the approach of refuting S-form DQBFs—which amounts to proving the non-existence of Skolem functions—by looking for Herbrand functions that may exist *even if the formula is true*.

The second difference is the absence of universal reduction. The difficulty of dealing with universal reduction in the context of DQBF resolution is to some extent addressed in [7], where it is considered in the (closely related [8]) context of dependency schemes. There it is shown that the interplay between universal reduction and merging is problematic, and additional constraints must be placed on universal reduction to prevent unsound inferences. Given that universal reduction is not necessary for completeness, it seems natural to dispense with it entirely.

The third and final difference is the explicit representation of functions in $\mathsf {M{\hbox {{-}}}Res}$, versus the function placeholders known as ‘merged literals’ from classical long-distance Q-resolution. Here we argue that the ‘full binary tree’ construction that features in the proofs of Lemmata 5 and 35 is the canonical completeness proof for CDCL-based systems. The explicit representation of functions is key to this construction, since it allows the comparison of non-trivial merge maps. Thus we argue that building strategies *into* proofs is the natural way to overcome incompleteness.

### Relevance to solving

Merge maps may be relevant for QBF and DQBF solving.

In dependency learning for QBF [45], variable dependencies are ignored until clause learning is blocked by an illegal merge. Our work demonstrates that many ‘illegal’ merges are perfectly sound inferences; moreover, $\mathsf {M{\hbox {{-}}}Res}$ provides a mechanism for identifying such cases based on isomorphism. Thus, it is plausible that incorporating merge maps could increase the scope of dependency learning.

In DQBF, practitioners are still looking for a natural ‘CDCL-based’ (as apposed to ‘expansion-based’) solving paradigm. Our discussion in Sect. 6 suggests one possible reason: namely, the use of Skolem form encodings is not conducive to CDCL-based search. An interesting direction for future work, therefore, would be to experiment with Herbrand-form DQBFs as the standard input format for CDCL-based DQBF solving.

It seems natural, then, to suggest Merge Resolution as the underlying resolution engine in a CDCL-based solver for Herbrand-form DQBF. Conceiving such an implementation would require some work; for example, one would need to store partial strategies with learned clauses, and carry out an efficient isomorphism test. Isomorphism is an easy way to determine the equivalence of two Boolean functions, but in general it seems unlikely that two equivalent functions will have identical representations. This points towards efficient (approximate) equivalence testing as the key to a successful implementation of $\mathsf {M{\hbox {{-}}}Res}$.

### Complexity of H-form DQBF

Whereas the decision problem for S-form DQBF is known to be $\mathsf {NEXP}$ complete [1], the complexity of the decision problem for H-form DQBFs, as far as we are aware, has not been studied. Moreover, the methodology of [1] does not seem appropriate for H-form DQBFs. Since every QBF can be written as an H-form DQBF, the decision problem is certainly $\mathsf {PSPACE}$-hard, and the NEXP upper bound applies for all DQBFs, but its exact complexity remains an interesting open problem.

## Footnotes

1 There exists associated proof systems for true QBFs [30].

2 Previous DQBF resolution systems either use expansion [13] or extension variables [50].

3 The notion is evident to a greater or lesser degree in all of the papers [4, 7, 23, 44, 46, 54].

4 Reductionless $\mathsf {LD{\hbox {{-}}}Q{\hbox {{-}}}Res}$ *p*-simulates level-ordered $\mathsf {Q{\hbox {{-}}}Res}$ by means of a simple construction, and is exponentially separated by the equality formulas [9]. It is also known that reductionless $\mathsf {LD{\hbox {{-}}}Q{\hbox {{-}}}Res}$ and $\mathsf {Q{\hbox {{-}}}Res}$ are incomparable [47].
